# PRMT5-Mediated Arginine Methylation of ACSL4 Attenuates Its Stability and Suppresses Ferroptosis in Renal Cancer

**DOI:** 10.34133/research.0789

**Published:** 2025-08-01

**Authors:** Meng Zhang, Hao Liu, Rong Yin, Jiayu Xu, Siqi Fan, Xingyou Qian, Menghan Cao, Shu Li, Ao Zhang, Guodong Chen, Hongmei Yong, Zhongwei Li, Jin Bai

**Affiliations:** ^1^ Cancer Institute, Xuzhou Medical University, Xuzhou, Jiangsu, China.; ^2^ Centre of Clinical Oncology, The Affiliated Hospital of Xuzhou Medical University, Xuzhou, Jiangsu, China.; ^3^ Department of Oncology, The Affiliated Huai’an Hospital of Xuzhou Medical University and The Second People’s Hospital of Huai’an, Huai’an, Jiangsu, China.; ^4^ Laboratory of Epigenetic Regulation in Molecular Medicine, School of Basic Medical Sciences, Wannan Medical College, Wuhu, Anhui, China.; ^5^ Anhui Province Key Laboratory of Basic Research and Transformation of Age-Related Diseases, Wannan Medical College, Wuhu, Anhui, China.; ^6^ Jiangsu Center for the Collaboration and Innovation of Cancer Biotherapy, Cancer Institute, Xuzhou Medical University, Xuzhou, Jiangsu, China.

## Abstract

Lipid-peroxidation-driven ferroptosis represents a vital mode of regulated cell death increasingly recognized for its role in cancer therapy. Lipid metabolism plays a crucial role in determining the vulnerability of cells to ferroptosis, particularly in cancer cells. The biosynthesis and remodeling of polyunsaturated fatty acid–phosphatidylethanolamine in cell membranes rely heavily on the activity of acyl-CoA synthetase family member 4 (ACSL4). However, the regulatory mechanisms governing ACSL4 expression in renal cell carcinoma (RCC) remain unclear. In this study, we screened a library of 765 epigenetic compounds to identify novel regulators of ferroptosis. Notably, we discovered that protein arginine methyltransferase 5 (PRMT5) inhibitors markedly promote ferroptosis in RCC cells. Inhibition or knockdown of PRMT5 in RCC cell lines enhanced lipid peroxidation and reduced cell viability. PRMT5 interacted with and catalyzed the symmetric dimethylation of ACSL4 at the arginine 549 (R549) site, facilitating its degradation via the proteasome. In vivo, the combination of a PRMT5 inhibitor with anti-PD-1 therapy notably increased ferroptosis and reduced tumor growth. Furthermore, elevated PRMT5 levels were associated with unfavorable clinical outcomes in patients with renal cancer. Overall, our findings suggest that PRMT5 regulates ferroptosis in RCC by methylating ACSL4 at the R549 site, and its inhibition enhances the therapeutic efficacy of immunotherapy through the induction of ferroptosis.

## Introduction

The incidence of renal cell carcinoma (RCC), a frequently diagnosed urinary system malignancy surpassed only by prostate and bladder cancers, has been progressively rising in recent years [1,2]. RCC often presents with an insidious onset, with approximately 30% of patients already exhibiting regional organ or distant lymph node metastasis upon diagnosis [3]. The limited treatment options frequently lead to a poor prognosis. Consequently, identifying effective diagnostic and therapeutic targets for RCC is essential for improving patient outcomes.

Ferroptosis is a recently identified form of cell death that differs from autophagy and apoptosis, mainly driven by lipid peroxidation [4–7]. This process is often accompanied by changes in mitochondria, including reduced cristae, a ruptured outer membrane, and mitochondrial shrinkage and collapse [8]. Lipid peroxidation typically occurs when polyunsaturated fatty acids (PUFAs) are embedded within phospholipids (PUFA-PLs) and interact with the phospholipid bilayer [9]. Cells enriched in PUFA-PLs exhibit increased vulnerability to lipid peroxidation [10]. Long-chain acyl-CoA synthetase family member 4 (ACSL4) and lysophosphatidylcholine acyltransferase 3 (LPCAT3) are key drivers in the ferroptosis process [11]. ACSL4 facilitates ferroptosis by binding long-chain PUFAs, particularly arachidonic acid (20:4) and adrenic acid (22:4), to coenzyme A and incorporating them into plasma membrane phospholipids via LPCAT3 [12–14].

Protein arginine methyltransferase 5 (PRMT5) belongs to the type II subclass of the PRMT family [15]. PRMT5 catalyzes the formation of symmetric dimethylation (SDMA) by transferring 1 or 2 methyl groups from *S*-adenosylmethionine to the guanidino nitrogen of protein arginine, thereby influencing tumor progression through multiple pathways [16–18]. PRMT5 promotes various aspects of malignant cancer progression by dimethylating both histone and nonhistone substrates [17,19]. Recently, our study also found that PRMT5-mediated arginine methylation of ALKBH5 promotes immune escape in colorectal cancer [20]. However, it is still not fully understood how PRMT5 influences the progression of renal cancer, particularly its potential involvement in the ferroptosis of renal cancer cells.

In the present work, we conducted a screening of an epigenetic compound library and identified several PRMT5 inhibitors (PRMT5is) that induce ferroptosis in RCC cells. We confirm that PRMT5 suppresses the progression of ferroptosis in RCC cells both in vitro and in vivo. Regarding the underlying mechanism, we demonstrate that PRMT5 binds to ACSL4 and reduces its expression levels. Additionally, we show that PRMT5 promotes the binding of ACSL4 to UBR5 via arginine 549 (R549) methylation, thereby inhibiting ferroptosis in renal cancer cells. Furthermore, combining a PRMT5i (GSK3326595) with PD-1 blockade markedly improves the therapeutic effectiveness of immunotherapy in vivo. Our findings uncover a possible molecular mechanism by which PRMT5 modulates ACSL4 expression to control ferroptosis and underscore the considerable potential of GSK3326595 as a novel antitumor agent.

## Results

### PRMT5 suppresses ferroptosis in renal cancer cells

**Fig. 1.**
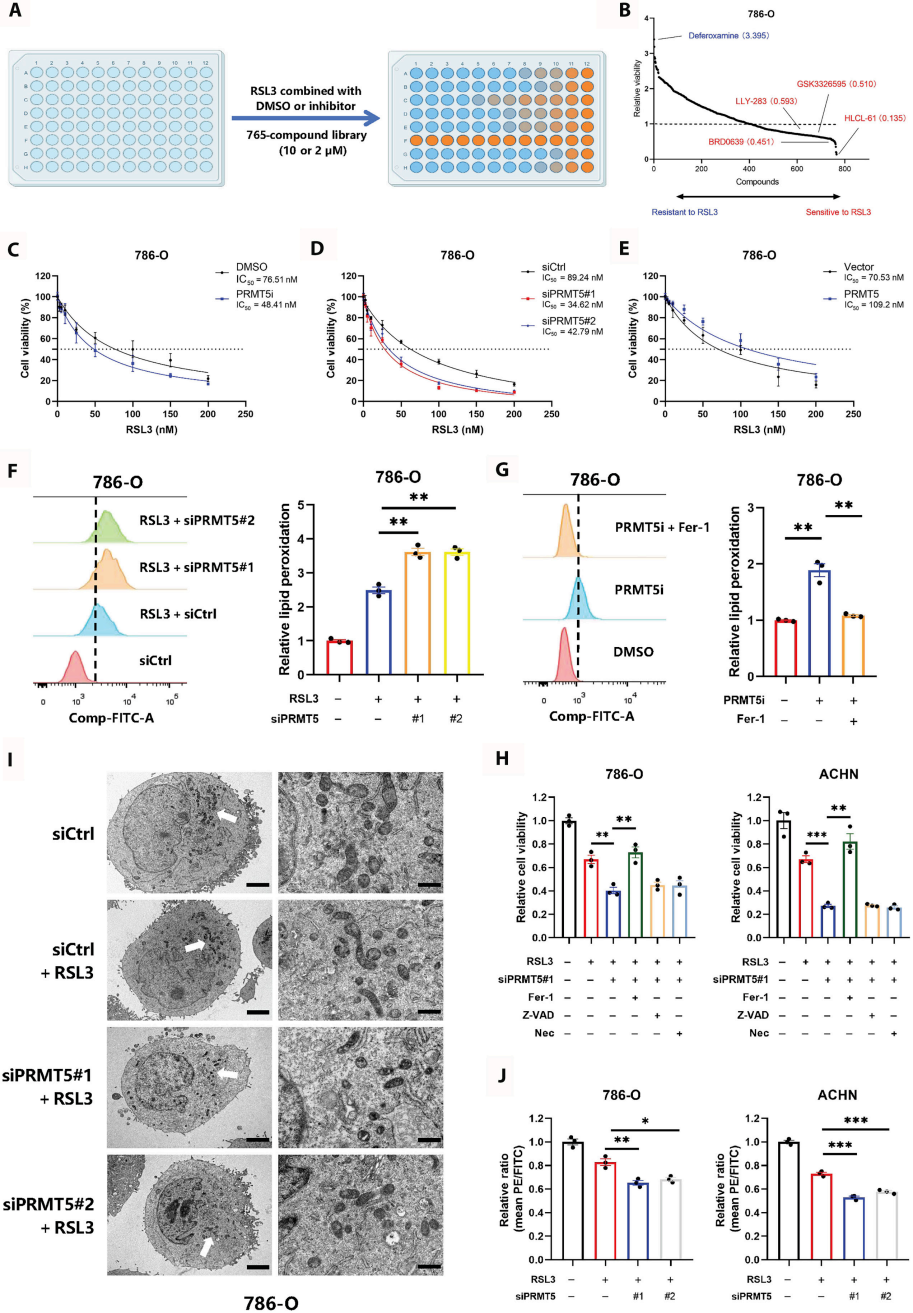
PRMT5 inhibits ferroptosis in renal cancer cells. (A) Schematic of the epigenetic compound library screening strategy. Illustrations were created with BioRender. (B) 786-O cells were seeded into 96-well plates for 24 h. Following treatment with 0.2 μM RSL3 combined with DMSO or 10 μM inhibitor, cell viability was assessed by the Cell Counting Kit-8 (CCK-8) assay. (C to E) After treatment with a PRMT5 inhibitor (GSK3326595) (C), PRMT5 knockdown (D), or overexpression (E), 786-O cells were treated with RSL3 for 12 h. The cell viability was examined using the CCK-8 assay. (F) PRMT5 knockdown 786-O cells were treated with 0.2 μM RSL3 for 12 h, and then BODIPY 581/591 C11 staining was performed to assess the lipid peroxidation. (G) 786-O cells were treated with 10 μM PRMT5 inhibitor combination with/without Fer-1 for 12 h, and then BODIPY 581/591 C11 staining was performed to assess the lipid peroxidation. (H) After 12 h of treatment with 0.2 μM RSL3 in combination with 1 μM Fer-1, 10 μM Z-VAD, or 10 μM necrostatin-1, the cell viability of PRMT5 knockdown 786-O and ACHN cells was detected by CCK-8 assay. (I) Transmission electron microscopy (TEM) images of the 786-O cells treated with RSL3 (0.2 μM) for 12 h. The white arrows point to mitochondria. Scale bars: left, 5 μm; right, 1 μm. (J) 786-O and ACHN cells were used to assess mitochondrial membrane potential by JC-1 staining. *P < 0.05; **P < 0.01; ***P < 0.001; NS, no significance. PRMT5, protein arginine methyltransferase 5; DMSO, dimethyl sulfoxide; PRMT5i, PRMT5 inhibitor; siCtrl, small interfering RNA control; siPRMT5, small interfering RNA targeting PRMT5; Fer-1, ferrostatin-1; Nec, necrostatin-1; PE, phosphatidylethanolamine; Comp-FITC-A, compensated fluorescein isothiocyanate–area.

To identify potential epigenetic compounds involved in ferroptosis, we screened an epigenetic compound library consisting of 765 compounds using an RSL3-induced ferroptosis model in 786-O cells (Fig. 1A). This screening revealed several PRMT5is that significantly enhanced RSL3-induced ferroptosis (Fig. 1B). The role of PRMT5 in the process of ferroptosis was further examined in 786-O and ACHN renal cancer cell lines. Following RSL3 induction, the cells were either treated with PRMT5is or subjected to PRMT5 knockdown for 24 h. Compared to that in the control groups, cell viability was markedly reduced in the PRMT5-inhibited or PRMT5-silenced groups (Fig. 1C and D and Fig. S1A, B, and L). Conversely, PRMT5 overexpression significantly increased cell viability (Fig. 1E and Fig. S1C).

To further elucidate the relationship between PRMT5 and ferroptosis, we assessed lipid peroxidation levels using BODIPY-581/591 staining and malondialdehyde (MDA) assay. Our findings showed that PRMT5 knockdown significantly increased the lipid peroxidation levels induced by RSL3 or erastin (Fig. 1F and Fig. S1D and G), whereas overexpression of PRMT5 exhibited the opposite effect on peroxidation and MDA levels (Fig. S1E to F, H to J, and N to O). Furthermore, treatment with PRMT5is alone markedly elevated lipid peroxidation, an effect that was reversed by ferrostatin-1 (Fer-1) (Fig. 1G and Fig. S1K and M).

To evaluate the role of PRMT5 in ferroptosis within RCC cells, we administered inhibitors targeting various forms of cell death. The findings revealed that silencing PRMT5 markedly increased cell death triggered by RSL3, an effect that was notably prevented by the ferroptosis inhibitor Fer-1 but not by the apoptosis inhibitor Z-VAD or the necroptosis inhibitor necrostatin-1 (Fig. 1H). Mitochondrial alterations, a hallmark of ferroptosis, were observed using transmission electron microscopy (TEM). Silencing PRMT5 led to a reduction in mitochondrial cristae, resulting in noticeable shrinkage and collapse (Fig. 1I). Furthermore, the mitochondrial membrane potential was assessed using the JC-1 assay. Cells with PRMT5 knockdown exhibited decreased red fluorescence and increased green fluorescence, indicating a reduction in mitochondrial membrane potential, which is consistent with the TEM observations (Fig. 1J). Collectively, these findings suggest that PRMT5 directly suppresses ferroptosis in RCC cells in vitro.

### PRMT5 binds to and inhibits the expression of ACSL4 in renal cancer cells

**Fig. 2.**
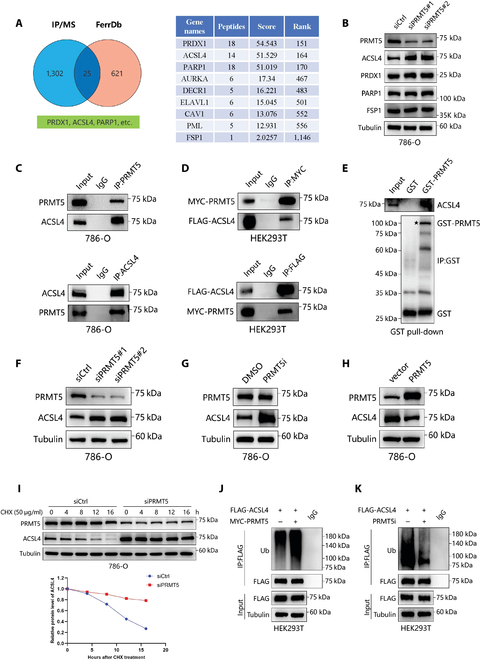
PRMT5 binds to and inhibits acyl-CoA synthetase family member 4 (ACSL4) expression in renal cancer cells. (A) Screening strategy for identifying the possible substrate of PRMT5 in ferroptosis. (B) The expressions of possible substrates of PRMT5 in ferroptosis at protein levels were examined by western blot in 786-O cells. (C) IP assays were conducted to detect the endogenous interaction between PRMT5 and ACSL4 in 786-O cells. (D) IP assays were conducted to detect the exogenous interaction between PRMT5 and ACSL4 by using antibodies against MYC and FLAG in HEK293T cells. (E) Purified glutathione *S*-transferase (GST)-tagged PRMT5 was pulled down with the 786-O cell lysates (asterisk: position of GST-PRMT5). (F to H) ACSL4 protein levels were analyzed by western blot in 786-O cells treated by PRMT5 knockdown (F), a PRMT5 inhibitor, (G) or overexpressing PRMT5 (H). (I) The ACSL4 protein expression was examined by western blot in 786-O-siCtrl and 786-O-siPRMT5 cells following treatment by cycloheximide (CHX; 50 μg/ml) for the specified time. (J and K) Western blots were performed to detect FLAG-ACSL4-associated ubiquitination after IP FLAG-Ub in PRMT5 upregulation cells (J) and PRMT5 inhibition cells (K). MS, mass spectrometry; IP, immunoprecipitation; IgG, immunoglobulin G.

To investigate how PRMT5 inhibits ferroptosis in RCC, we proposed that PRMT5 may influence ferroptosis by altering intracellular iron homeostasis. We measured intracellular divalent iron (Fe^2+^) levels, and the results showed no significant changes in Fe^2+^ levels upon PRMT5 knockdown (Fig. S2N and O). Therefore, we further explored the potential mechanism by which PRMT5 regulates ferroptosis in RCC. We conducted a mass spectrometry (MS) analysis of PRMT5-binding proteins to explore the molecular pathway through which PRMT5 suppresses ferroptosis in renal cancer cells (Fig. S2M). By comparing the MS results with potential ferroptosis-related proteins from the FerrDb database, we identified 25 candidate substrates that may be regulated by PRMT5 in the context of ferroptosis (Fig. 2A). Among the top candidates, only the expression of ACSL4 increased following PRMT5 knockdown, while the expression levels of other substrates remained unchanged (Fig. 2B). We subsequently performed coimmunoprecipitation (Co-IP) experiments, which revealed an endogenous interaction between PRMT5 and ACSL4 (Fig. 2C). To further confirm this interaction, HEK293T cells were cotransfected with MYC-tagged PRMT5 and FLAG-tagged ACSL4 plasmids, followed by Co-IP analysis, which confirmed the exogenous binding between the 2 proteins with consistent outcomes (Fig. 2D). Additionally, glutathione *S*-transferase (GST) pull-down experiments confirmed the binding between GST-PRMT5 and ACSL4 (Fig. 2E). Collectively, our data demonstrate that PRMT5 directly binds to ACSL4 and inhibits its expression in RCC cells.

We expanded our investigation into the role of PRMT5 in ACSL4 expression. The knockdown of PRMT5 and treatment with its inhibitor significantly increased ACSL4 protein levels; however, no changes were observed at the transcriptional level (Fig. 2F and G and Fig. S2A to I). In contrast, the overexpression of PRMT5 inhibited ACSL4 expression (Fig. 2H). We hypothesized that PRMT5 might influence ACSL4 stability through the ubiquitin–proteasome pathway and tested this hypothesis by treating 786-O cells with the protein synthesis inhibitor cycloheximide (CHX). Following CHX treatment, the half-life of ACSL4 was significantly prolonged in cells with PRMT5 knockdown and those treated with the PRMT5i GSK3326595 (Fig. 2I and Fig. S2J).

Since both the proteasomal and lysosomal pathways can mediate protein degradation, we treated the cells with the proteasome inhibitor MG132 and the autophagy receptor inhibitor chloroquine. MG132 effectively reversed the degradation of ACSL4 protein in cells overexpressing PRMT5, whereas chloroquine treatment did not affect ACSL4 levels (Fig. S2K and L). Additionally, we confirmed that PRMT5 enhances the ubiquitination of ACSL4 (Fig. 2J and K). The findings suggest that PRMT5 binds to ACSL4 and suppresses its stability through the proteasomal pathway.

### PRMT5 mediates the symmetric dimethylation of ACSL4 at the R549 site

**Fig. 3.**
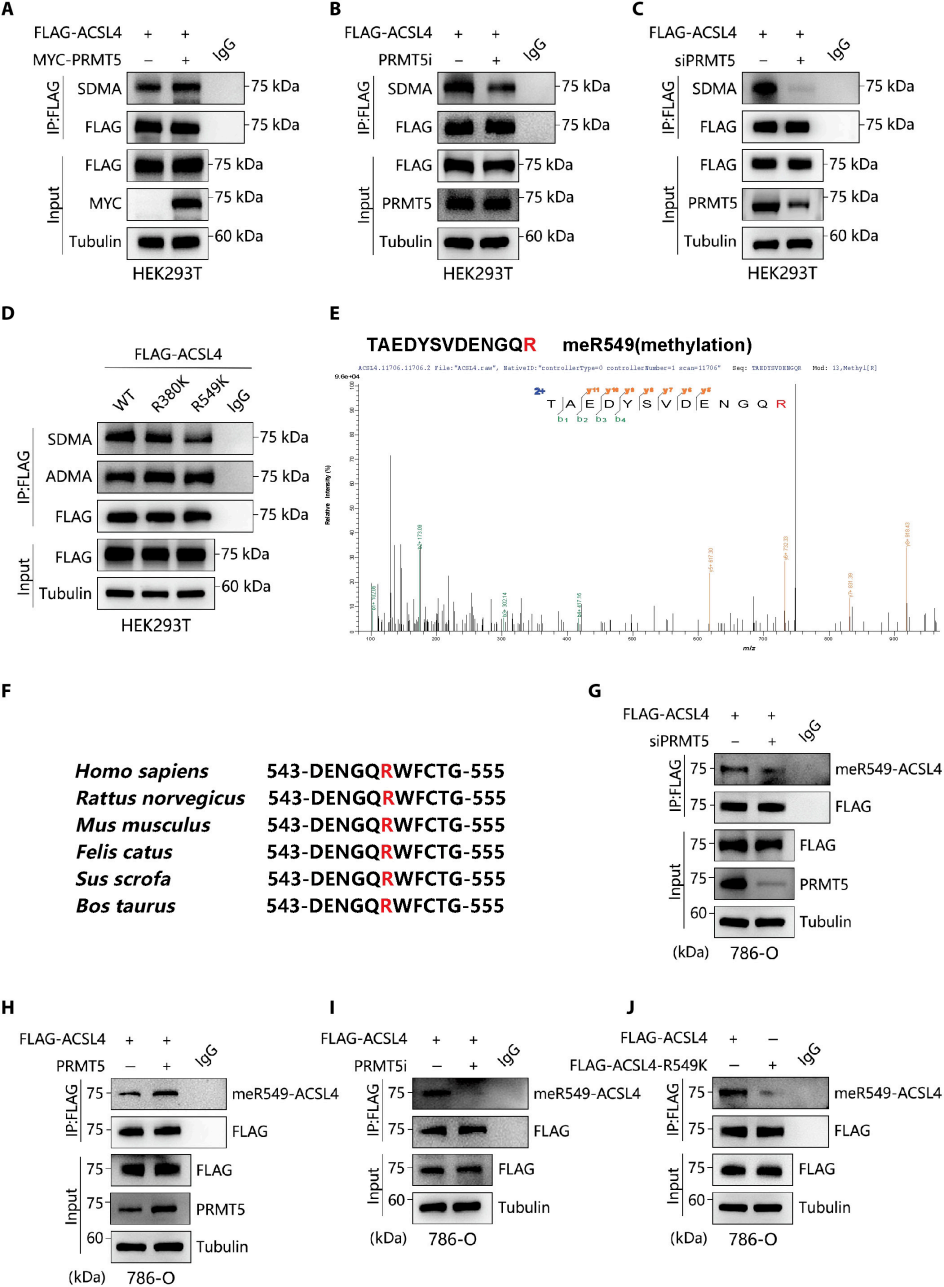
PRMT5 mediates symmetric dimethylation (SDMA) formation at arginine 549 (R549) of ACSL4. (A to C) Co-IP was conducted to examine the SDMA levels of ACSL4 with PRMT5 upregulation (A), inhibition (B), or knockdown (C). (D) Co-IP was conducted to test the SDMA changes in wild-type (WT) ACSL4 and R380K and R549K mutants. (E) The secondary mass spectrometry analysis identified a potential methylation site at R549. (F) The ACSL4 R549 site amino acid in different species. (G to J) Co-IP was performed to detect the meR549-ACSL4 levels with PRMT5 deficiency (G), upregulation (H), inhibition (I), or R549K mutant (J). Co-IP, co-immunoprecipitation.

As PRMT5 is a methyltransferase, previous studies have demonstrated that PRMT5-mediated arginine methylation of its substrates can regulate the protein stability of these substrates. Building on our earlier findings, we hypothesized that PRMT5 attenuates ACSL4 expression by catalyzing the methylation of arginine residues in ACSL4. To test this hypothesis, we conducted immunoprecipitation (IP) experiments, which revealed that the overexpression of PRMT5 was associated with elevated levels of SDMA in ACSL4 (Fig. 3A). In contrast, treatment with the PRMT5i GSK3326595 and knockdown of PRMT5 resulted in a reduction of SDMA levels in ACSL4 (Fig. 3B and C).

We conducted an MS analysis of ACSL4 protein modifications to identify specific methylation sites, which revealed 2 potentially methylated arginine residues: R380 and R549 (Fig. 3D and Fig. S3A and B). Subsequently, we constructed 2 mutant plasmids with R-to-K mutations and transfected them into HEK293T cells to evaluate the methylation status of ACSL4. The findings indicated that only the R549K mutation significantly reduced the levels of SDMA in ACSL4, while asymmetric dimethylation (ADMA) levels remained unaffected (Fig. 3D). Notably, the R549 site is highly conserved across various species (Fig. 3F), underscoring its functional significance.

Additionally, we synthesized and purified a specific antibody against the SDMA of ACSL4 at the R549 position (anti-meR549-ACSL4). This antibody specifically recognized ACSL4-R549 symmetric dimethylated peptides in a dot blot assay (Fig. S3C and D). IP experiments demonstrated a positive correlation between PRMT5 expression and the levels of meR549-ACSL4 (Fig. 3G and H). Furthermore, treatment with the PRMT5i GSK3326595 reduced the enrichment of ACSL4-R549me2s (Fig. 3I). Additionally, the ACSL4-R549K mutation inhibited the enrichment of ACSL4-R549me2s (Fig. 3J). In summary, our results indicate that PRMT5 symmetrically dimethylates ACSL4 at the R549 site.

### PRMT5-mediated meR549-ACSL4 decreases ACSL4 stability by enhancing the interaction between ACSL4 and UBR5

**Fig. 4.**
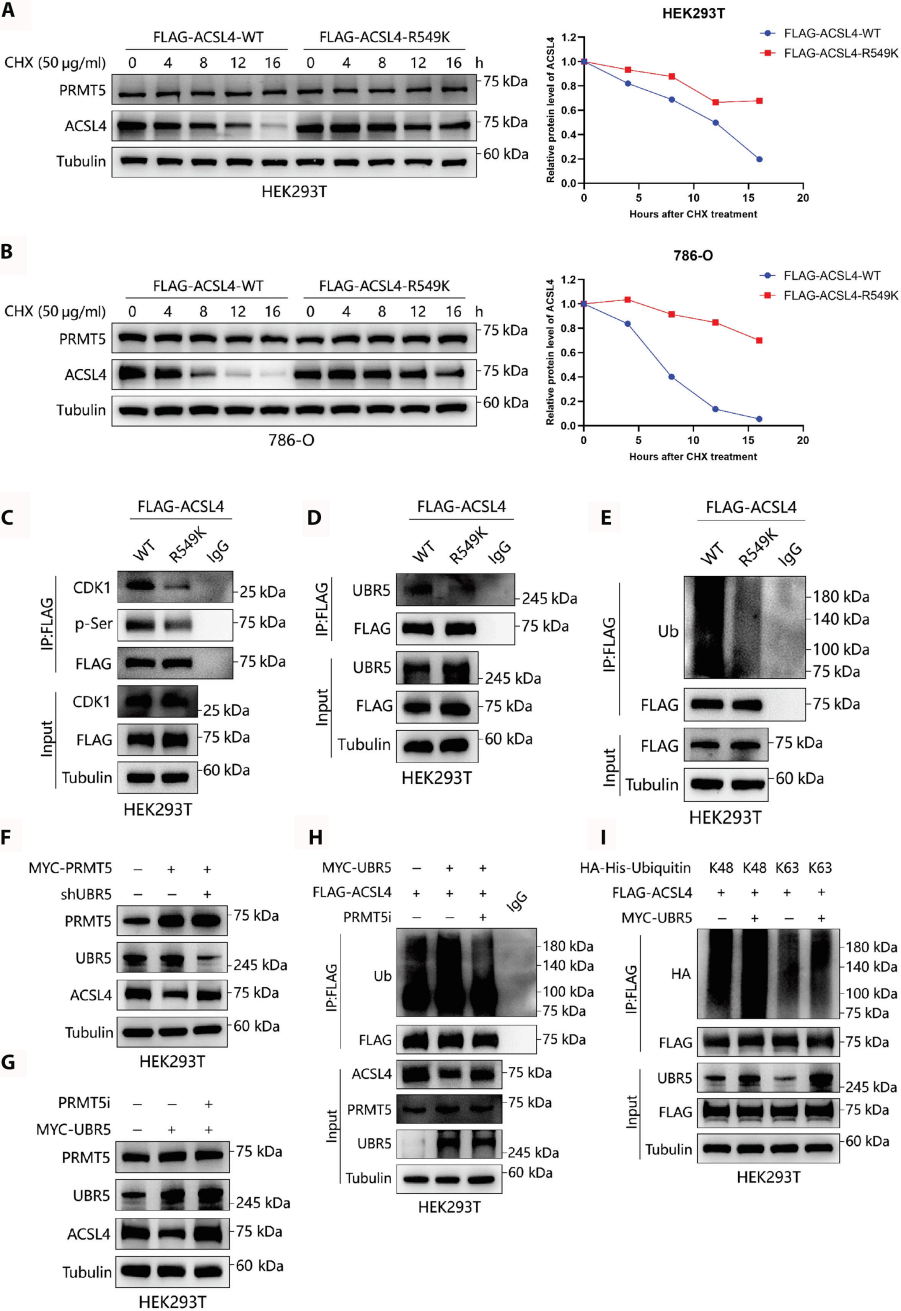
PRMT5-mediated meR549-ACSL4 decreases ACSL4 stability by promoting the binding of UBR5 with ACSL4. (A and B) The ACSL4 protein expression was examined by western blot in ACSL4-WT and ACSL4-R549K mutant HEK293T (A) and 786-O (B) cells following treatment by CHX (50 μg/ml) for the specified time. (C) Co-IP was performed to detect ACSL4 interaction with CDK1 and the serine phosphorylation levels in ACSL4-WT and ACSL4-R549K mutant HEK293T cells. (D) Co-IP was performed to detect ACSL4 interaction with UBR5 in ACSL4-WT and ACSL4-R549K mutant HEK293T cells. (E) Western blots were performed to detect FLAG-ACSL4-associated ubiquitination after IP FLAG-Ub in ACSL4-WT cells and ACSL4-R549K mutant cells. (F) Western blot analysis of HEK293T cells transfected with the indicated plasmid. The protein levels of PRMT5, UBR5, and ACSL4 were detected. (G) Western blot analysis of vectors and UBR5 upregulation HEK293T cells treated with DMSO or the PRMT5 inhibitor for 24 h. The protein levels of PRMT5, UBR5, and ACSL4 were detected. (H) Western blots were conducted to detect FLAG-ACSL4-associated ubiquitination after IP FLAG-Ub in HEK293T cells treated with or without the PRMT5 inhibitor for 24 h. (I) HEK293T cells were transfected with the FLAG-ACSL4 plasmid and HA-His-ubiquitin K48 or HA-His-ubiquitin K63 plasmids, either alone or together with the UBR5-MYC plasmid. Western blotting was then performed to detect FLAG-ACSL4-associated ubiquitination.

To investigate whether ACSL4-R549 methylation affects the stability of ACSL4, we stably expressed either the wild-type ACSL4 (ACSL4-WT) or the ACSL4-R549K mutant proteins in HEK293T and 786-O cells treated with CHX. Our results demonstrated that the R549K mutant significantly prolonged the half-life of the ACSL4 protein (Fig. 4A and B), indicating that PRMT5-mediated methylation of ACSL4-R549 promotes its degradation.

In our previous result, we demonstrated that PRMT5 mediates the ubiquitination of ACSL4 via the proteasomal pathway. We proposed that the methylation of ACSL4 at the R549 position enhances the interaction between ACSL4 and the ubiquitin ligase. Previous research has shown that CDK1 increases the binding of the E3 ligase UBR5 to ACSL4 by phosphorylating ACSL4 at the S447 residue [21]. Given that the S447 and R549 residues of ACSL4 are in close proximity, we hypothesized that the dimethyl group at the R549 site may induce a conformational change that facilitates the CDK1 phosphorylation of S447, thereby promoting ACSL4-S447 phosphorylation. Therefore, we propose that the PRMT5-mediated methylation of ACSL4 at R549 enhances the CDK1-mediated phosphorylation of ACSL4 at S447, leading to enhanced binding of UBR5 and the subsequent degradation of ACSL4.

In order to verify this hypothesis, we conducted Co-IP analysis in both ACSL4-WT and ACSL4-R549K cells to determine whether the methylation of ACSL4-R549 influences the interaction between ACSL4 and CDK1, as well as the phosphorylation of ACSL4 by CDK1. The results indicated that the interaction between ACSL4 and CDK1 was diminished following the ACSL4-R549 mutation, accompanied by a reduction in the serine phosphorylation level of ACSL4 in both HEK293T and 786-O cells (Fig. 4C and Fig. S4A). Similar findings were observed in 786-O cells when PRMT5 was knocked down, which did not impact CDK1 expression (Fig. S4B). Moreover, treatment with the CDK1 inhibitor Ro-3306 significantly attenuated the PRMT5-mediated degradation of ACSL4, indicating that CDK1 activity is indeed involved in this process (Fig. S4C).

To determine whether ACSL4-R549 methylation influences the binding of ACSL4 to UBR5 and its subsequent ubiquitination, we conducted Co-IP experiments. The results demonstrated that the R549K mutation diminished the binding affinity of ACSL4 for UBR5 and inhibited the ubiquitination of ACSL4 (Fig. 4D and E). Furthermore, we explored the role of UBR5 in the PRMT5-mediated regulation of ACSL4 protein levels. Notably, the knockdown of UBR5 restored ACSL4 protein levels, while the inhibition of PRMT5 suppressed UBR5-mediated ubiquitination and degradation of ACSL4 (Fig. 4F to H). Given that UBR5 ubiquitinates methylated ACSL4, we further investigated the specific type of ubiquitination facilitated by UBR5. The findings revealed that UBR5 mediates the K48-linked polyubiquitination of ACSL4, as opposed to K63-linked polyubiquitination (Fig. 4I). In conclusion, our data suggest that ACSL4-R549 methylation is essential for the binding of UBR5 to ACSL4 and promotes the degradation of ACSL4.

### PRMT5 induces ferroptosis resistance in renal cancer cells by catalyzing ACSL4-R549 methylation

**Fig. 5.**
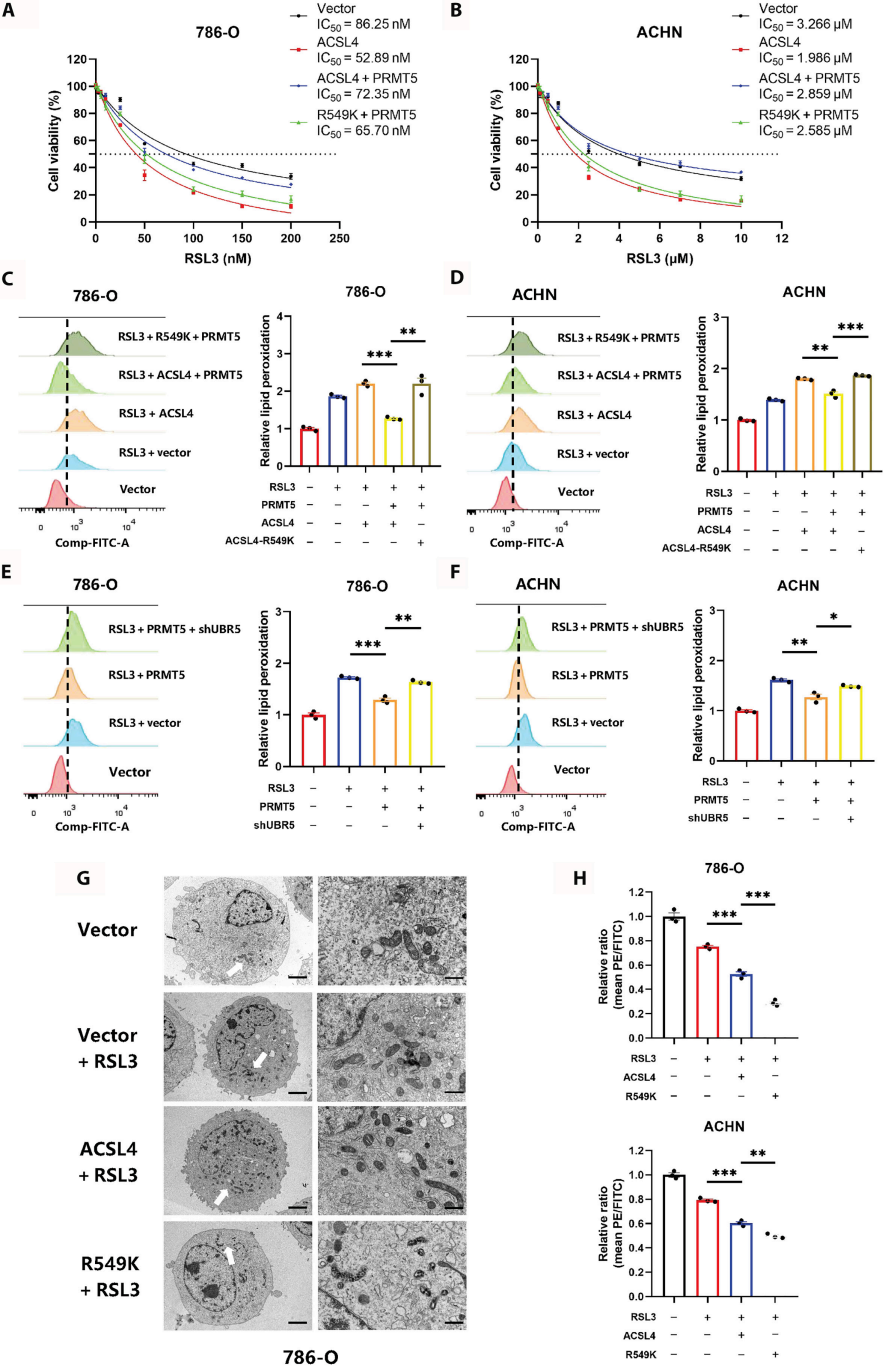
PRMT5 induces ferroptosis resistance in renal cancer cells by R549-ACSL4 methylation. (A and B) After treatment with overexpressing ACSL4, overexpressing ACSL4 combination of PRMT5, and overexpressing ACSL4-R549K combination of PRMT5, 786-O (A) and ACHN (B) cells were treated with RSL3 for 12 h. The cell viability was examined using the CCK-8 assay. (C and D) After treatment with overexpressing ACSL4, overexpressing ACSL4 combination of PRMT5, and overexpressing ACSL4-R549K combination of PRMT5, 786-O (C) and ACHN (D) cells were treated with RSL3 for 12 h. BODIPY 581/591 C11 staining was performed to assess the lipid peroxidation. (E and F) After treatment with overexpressing PRMT5 and overexpressing PRMT5 combination of UBR5 knockdown, 786-O (E) and ACHN (F) cells were treated with RSL3 for 12 h. BODIPY 581/591 C11 staining was performed to assess the lipid peroxidation. (G) TEM images of the 786-O cells treated with RSL3 (0.2 μM) for 12 h. The white arrows point to mitochondria. Scale bars: left, 5 μm; right, 1 μm. (H) 786-O and ACHN cells were used to assess the mitochondrial membrane potential by JC-1 staining. *P < 0.05; **P < 0.01; ***P < 0.001.

We demonstrated that PRMT5 methylates ACSL4 at the R549 site, which influences its protein stability. To further investigate the role of ACSL4-R549 methylation in ferroptosis, we established stable RCC cell lines expressing either wild-type ACSL4 (ACSL4-WT) or the mutant ACSL4-R549K. The results indicated that PRMT5 overexpression rescued ferroptosis in ACSL4-WT RCC cells but had minimal effect on cell viability in ACSL4-R549K cells (Fig. 5A and B and Fig. S5A and B). Similar findings were observed in lipid peroxidation assays (Fig. 5C and D). Furthermore, UBR5 knockdown significantly increased lipid peroxidation in RCC cells expressing PRMT5 (Fig. 5E and F and Fig. S5C and D).

To investigate the impact of ACSL4-R549 methylation on mitochondrial morphology in RCC cells, we conducted TEM. Our observations indicated that ACSL4 overexpression led to mitochondrial shrinkage, with ACSL4-R549K cells exhibiting even more pronounced shrinkage (Fig. 5G). The results of JC-1 staining corroborated the TEM findings, demonstrating that ACSL4-R549K overexpression significantly diminished mitochondrial membrane potential (Fig. 5H). These results suggest that ACSL4-R549 methylation suppresses ferroptosis in RCC cells.

### PRMT5-mediated meR549-ACSL4 formation correlates with poor prognosis in renal cancer patients

**Fig. 6.**
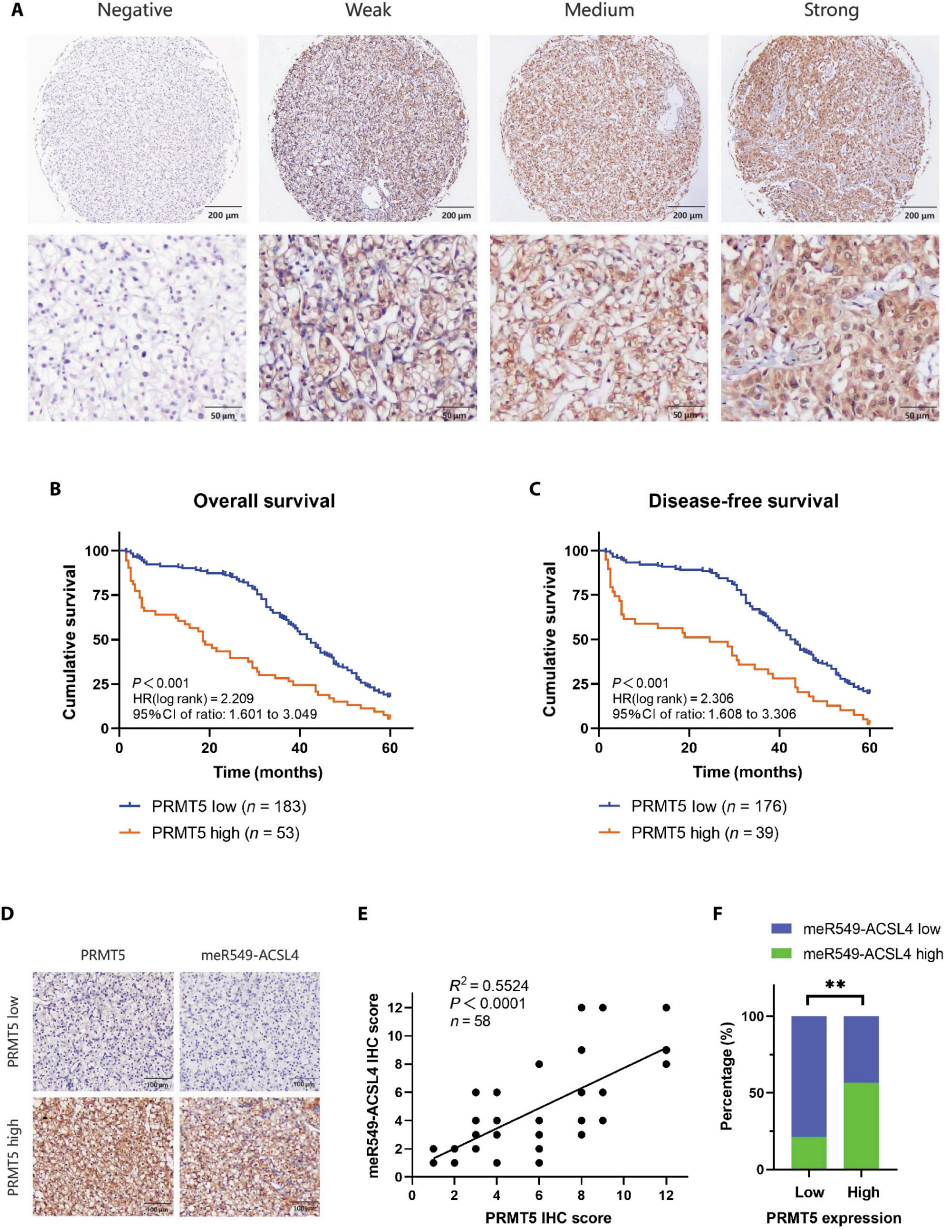
PRMT5-mediated meR549-ACSL4 formation correlates with poor prognosis in renal cancer patients. (A) Representative images of PRMT5 IHC staining in tissue microarray (TMA) are shown. (B) High PRMT5 expression correlated with a poorer 5-year overall survival for 236 renal cancer patients (*P* < 0.001, log-rank test). (C) High PRMT5 expression correlated with a poorer 5-year disease-free survival for 215 renal cancer patients (*P* < 0.001, log-rank test). (D) Representative images of PRMT5 and meR549-ACSL4 IHC staining in a PRMT5-low case and a PRMT5-high case in renal cancer patients are presented. (E) Correlation between PRMT5 and meR549-ACSL4 expression was detected by the Pearson correlation coefficient test. (F) Correlation between PRMT5 and meR549-ACSL4 expression was detected by the Pearson chi-square test. IHC, immunohistochemistry; HR, hazard ratio.

To investigate the role of PRMT5 in the progression of RCC in clinical patients, we analyzed PRMT5 protein expression in tissue microarray (TMA) sections from 286 RCC patients (Fig. 6A). Pearson chi-square tests were conducted to evaluate the correlation between PRMT5 expression and clinical–pathological features, as summarized in Table S1. The results indicated that the high PRMT5 expression in RCC patients was significantly linked to a tumor size greater than 7 cm (*P* = 0.002), a greater invasion depth (*P* < 0.001), lymph node metastasis (*P* = 0.005), and distant metastasis (*P* < 0.001). Additionally, Kaplan–Meier survival curves demonstrated that high PRMT5 expression was strongly associated with poor overall survival (OS) (*P* < 0.001) and disease-free survival (DFS) (*P* < 0.001) in RCC patients (Fig. 6B and C).

To evaluate whether PRMT5 expression serves as an independent predictor of prognosis in patients with RCC, we performed Cox regression analyses. The univariate analysis demonstrated that PRMT5 expression was markedly associated with OS and DFS in RCC patients (see Table S2). Multivariate analysis revealed that only PRMT5 expression and distant metastasis were significantly associated with prognosis, while age, tumor size, and lymph node metastasis showed no significant correlation. Notably, the multivariate analysis confirmed that PRMT5 expression is an independent predictor of prognosis for both OS (hazard ratio [HR] 1.665, 95% confidence interval [CI] 1.146 to 2.417, *P* = 0.007) and DFS (HR 1.845, 95% CI 1.229 to 2.759, *P* = 0.003) in this patient population (see Table S3).

To further investigate the relationship between PRMT5 expression and meR549-ACSL4 levels in RCC patients, we analyzed TMA sections from 58 RCC patients (Fig. 6D). Our analysis revealed a positive correlation between PRMT5 and meR549-ACSL4 levels (Fig. 6E). Furthermore, high PRMT5 expression was strongly associated with increased meR549-ACSL4 expression (Fig. 6F). These findings indicate that PRMT5 expression is notably correlated with the clinical prognosis of RCC patients and is positively associated with meR549-ACSL4 expression levels.

### The PRMT5i represses renal cell tumorigenesis and enhances the therapeutic effects of immunotherapy by promoting ferroptosis

**Fig. 7.**
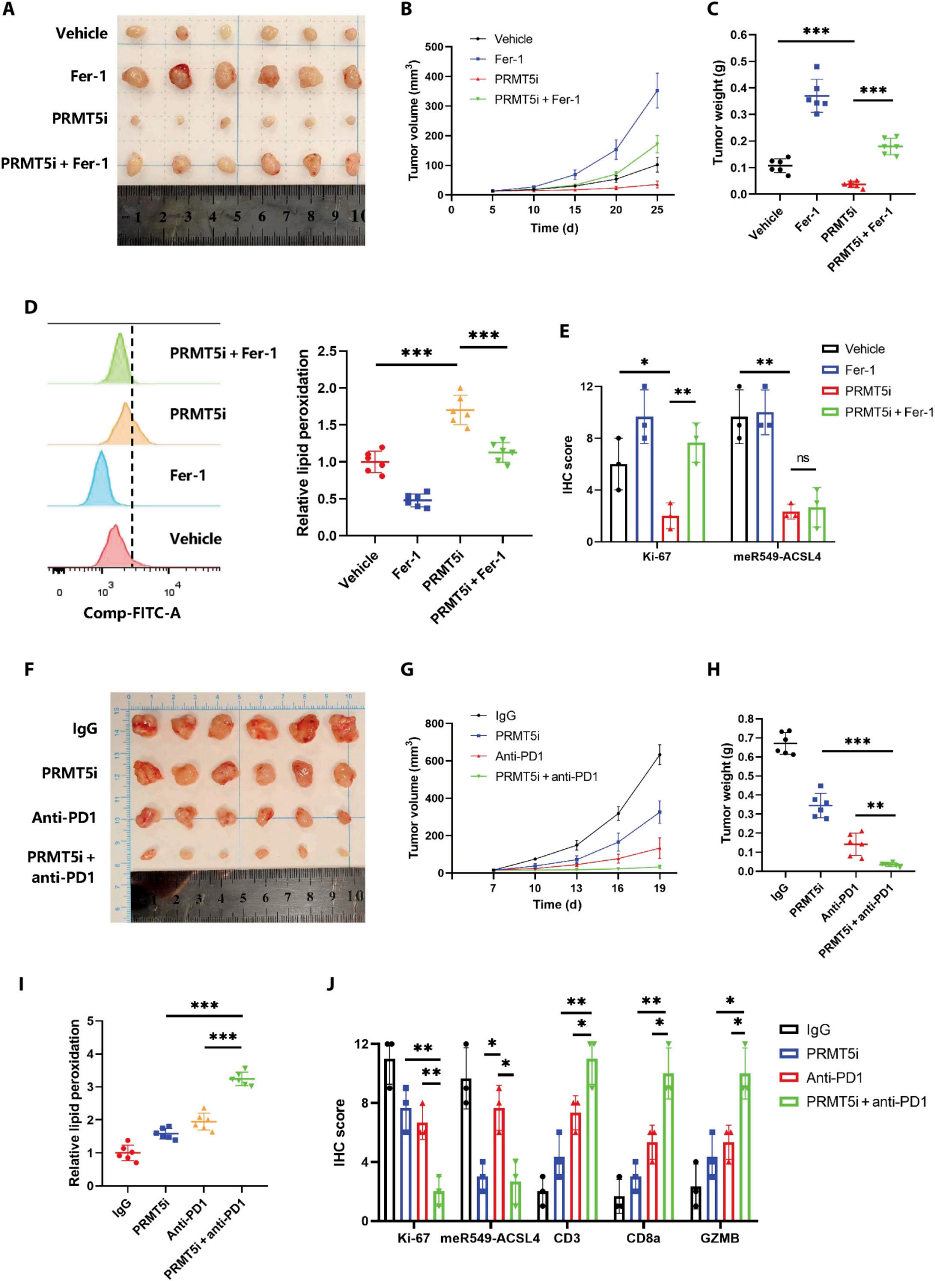
PRMT5 inhibitor enhances the therapeutic effect of immunotherapy by promoting ferroptosis. (A) Images of tumor size in different groups are shown. (B and C) The tumor volumes (*n* = 6) and tumor weights (*n* = 6) of the subcutaneous xenografts were calculated. (D) Relative lipid peroxidation in tumor cells isolated from xenografts. (E) Quantification of Ki-67 and meR549-ACSL4 staining intensity in xenograft tumors. (F to I) Representative images (F), tumor growth curves (G), tumor volume (H), and relative lipid peroxidation in tumor cells (I) of different treatments in each group. (J) Representative images of the IHC staining of Ki-67, meR549-ACSL4, CD3, CD8a, and granzyme B (GZMB) in the tumor xenografts. *P < 0.05; **P < 0.01; ***P < 0.001.

Our previous data demonstrated that PRMT5is promote ferroptosis in RCC cells in vitro. To further investigate the in vivo effects of PRMT5i (i.e., GSK3326595) on tumor ferroptosis, we subcutaneously transplanted 786-O cells into nude mice and performed intratumoral injections of RSL3 in all tumors (Fig. 7A). Tumor growth curves and weight measurements indicated that PRMT5i significantly inhibited tumor growth (Fig. 7B and C). Additionally, lipid peroxidation levels were significantly elevated in PRMT5i-treated tumors (Fig. 7D). Immunohistochemistry (IHC) analysis revealed that PRMT5i suppressed tumor growth by reducing Ki-67 expression (Fig. 7E and Fig. S7A).

Earlier research has shown that immunotherapy-activated CD8^+^ T cells enhance tumor cell sensitivity to ferroptosis, and this elevated ferroptosis plays a role in reinforcing the antitumor effects of immunotherapeutic strategies [22,23]. Therefore, we further investigated whether the PRMT5i promotes immunotherapy-induced ferroptosis in vivo. The mice were divided into 4 groups: the immunoglobulin G antibody control group, anti-PD-1 treatment group, PRMT5i treatment group, and combination of anti-PD-1 and PRMT5i treatment group (Fig. 7F). Compared to treatment with the anti-PD-1 antibody alone, the combination therapy group significantly reduced Renca cell tumor growth (Fig. 7G and H). Tumors from the group receiving the combination therapy exhibited a pronounced elevation in lipid peroxidation relative to the other treatment groups (Fig. 7I). Additionally, IHC revealed that the combination of anti-PD-1 and PRMT5i synergistically reduced Ki-67 levels in Renca tumor cells while increasing markers of tumor-infiltrating immune cells, including CD3, CD8a, and granzyme B (Fig. 7J and Fig. S7B). In summary, the combination of the PRMT5-specific inhibitor GSK3326595 with anti-PD-1 antibody markedly enhances the efficacy of immunotherapy in vivo and promotes ferroptosis in renal tumor cells.

## Discussion

The limited treatment options for advanced renal cancer present substantial management challenges. Although the combination of targeted therapy and immunotherapy has demonstrated remarkable efficacy in clinical practice, it also has certain limitations. A major challenge of combination therapy is the emergence of resistance, which necessitates further research to optimize its application and enhance patient survival benefits [24]. Hence, exploring novel molecular targets for use alongside immunotherapy represents a highly promising approach to addressing immune resistance [25].

**Fig. 8.**
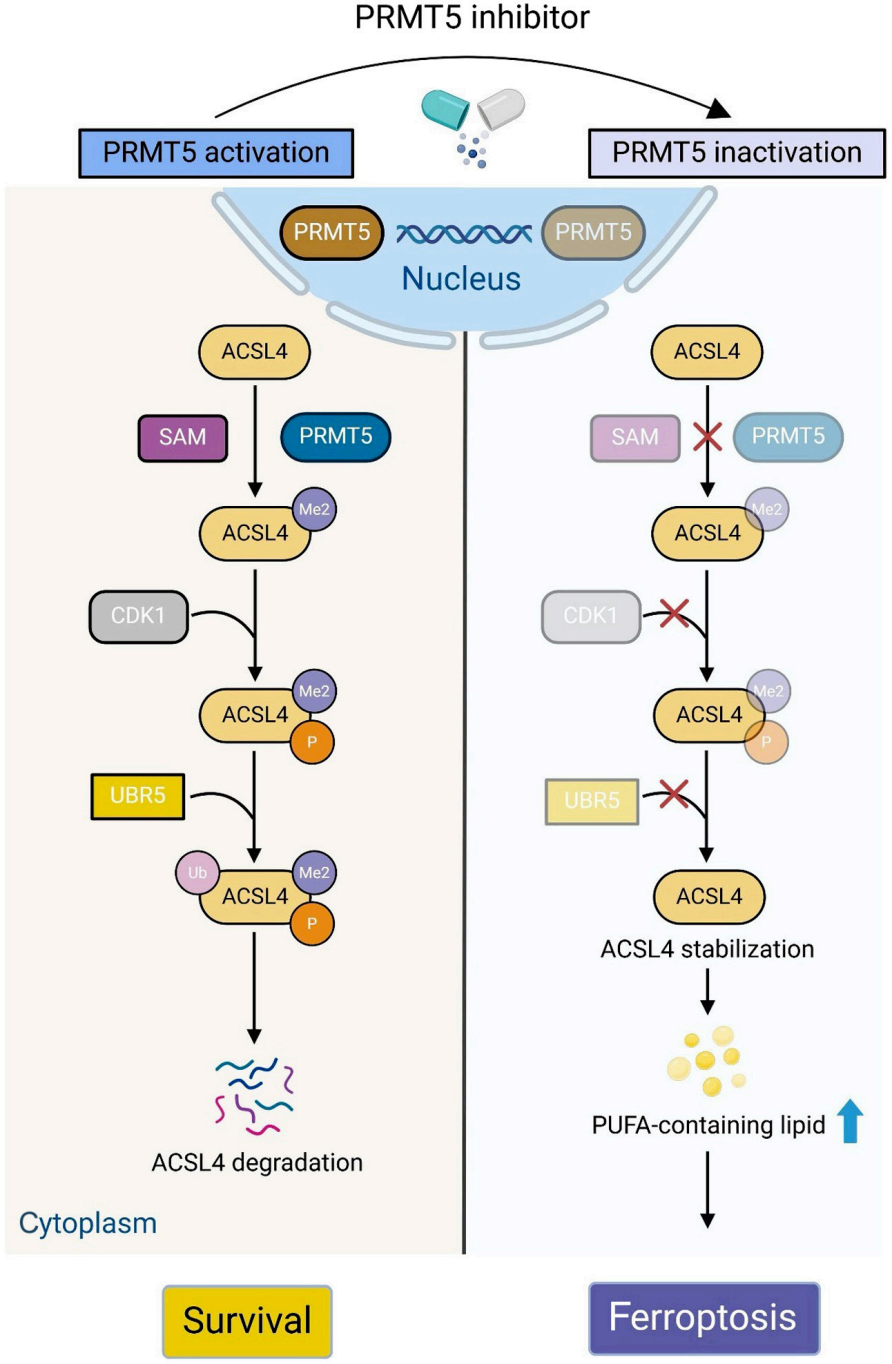
Schematic diagram of the hypothesized model for describing the role of PRMT5 inhibition-mediated methylation of ACSL4 in promoting ferroptosis in renal cancer. Illustrations were created with BioRender. SAM, *S*-adenosylmethionine; PUFA, polyunsaturated fatty acid.

This study demonstrated that PRMT5 inhibits ferroptosis in RCC cells, following the identification of the ferroptosis-promoting role of PRMT5is using an epigenetic compound library. Our findings confirmed that PRMT5 suppresses ferroptosis by binding to and methylating ACSL4 at the R549 residue. Furthermore, the methylation of ACSL4 enhances its interaction with UBR5, leading to UBR5-induced ubiquitination and degradation. This process, in turn, reduces lipid peroxidation and inhibits ferroptosis (Fig. 8A). These findings underscore the potential therapeutic value of inhibiting PRMT5 to enhance tumor cell ferroptosis and improve the efficacy of immunotherapy in renal cancer.

Multiple studies have confirmed that ACSL4 is a key factor in determining sensitivity to ferroptosis [11,26,27]. Hsp90 dephosphorylates Drp1 at the Ser637 site, promoting glioma ferroptosis by enhancing ACSL4-dependent lipid peroxidation [28]. Additionally, the phosphorylation of ACSL4 at the Thr328 site by PKCβII increases its activity, exacerbating ferroptosis [14]. Although the role of ACSL4 in regulating tumor ferroptosis is well-documented, its cooperative mechanisms with other proteins remain unclear. Notably, recent findings highlight the cross talk between arginine methylation and phosphorylation [29,30]. Our research has revealed for the first time that the interaction between meR549-ACSL4 and pS447-ACSL4 significantly reduces the stability of the ACSL4 protein. This methylation–phosphorylation interplay may help maintain cellular homeostasis and provide new insights into potential cancer drug combinations.

A report indicates that ACSL4 is methylated at the R339 site through ADMA modification by CARM1, which suppresses ferroptosis in colorectal cancer cells [31]. However, we discovered that ACSL4 is remarkably methylated by PRMT5 via SDMA methylation at the R549 site in RCC cells. These findings suggest that the R339 ADMA or R549 SDMA modifications of ACSL4 can regulate ferroptosis in tumor cells, highlighting the critical role of the arginine methylation of ACSL4 in this process. Increased levels of ferroptosis driven by ACSL4 enhance tumor sensitivity to PD-1 blockade therapy.

Interestingly, Fan et al.’s group [32] recently reported that PRMT5 can inhibit ferroptosis by mediating the methylation of GPX4 in cancer cells. In this study, the authors screened a CRISPR metabolic gene library and identified that MAT2A (methionine adenosyltransferase II alpha) may promote resistance to ferroptosis. Given the role of MAT2A in converting methionine to *S*-adenosylmethionine, they further validated that PRMT5 binds to and catalyzes the methylation of GPX4. In our research, we screened an epigenetic compound library and demonstrated that several PRMT5is enhance ferroptosis in cancer cells. Notably, among these PRMT5is, GSK3326595 is currently in phase II clinical trials, providing a new option and hope for cancer treatment [33,34]. Additionally, we have uncovered a novel mechanism in which PRMT5 methylates ACSL4 to regulate ferroptosis. Combined with our new findings, this suggests that PRMT5 plays a crucial role in suppressing ferroptosis in cancer cells and is expected to serve as a potential critical target for cancer therapy.

While our study strongly supports the role of PRMT5 in regulating ferroptosis in renal cancer, several areas warrant further investigation. First, although ACSL4 has been identified as a key substrate of PRMT5, other potential targets of PRMT5 in the regulation of ferroptosis remain unexplored. Future research should investigate the broader network of proteins modulated by PRMT5 in both ferroptosis and cancer progression. Second, the therapeutic potential of PRMT5 inhibition requires further validation through clinical trials, particularly in combination with immunotherapy, to confirm its translational relevance. Additionally, as PRMT5is have been evaluated in clinical trials for various tumors, it is crucial to assess their safety and efficacy in combination therapies for clinical application [35,36].

In summary, this study reveals a novel mechanism by which PRMT5 regulates ferroptosis in RCC cells, primarily through the symmetric dimethylation of ACSL4 at the R549 residue. This modification promotes the degradation of ACSL4, thereby protecting the cells from ferroptosis. Furthermore, the inhibition of PRMT5 sensitizes RCC cells to ferroptosis and enhances the efficacy of immunotherapy, presenting a potential therapeutic strategy for the treatment of RCC. The clinical association between PRMT5 expression, meR549-ACSL4 levels, and poor prognosis underscores the translational potential of targeting PRMT5 in renal cancer therapy.

## Materials and Methods

### Cell culture and treatment

Cell lines were sourced from the Cell Bank of the Chinese Academy of Sciences. Experimental procedures and treatment details are described in the Supplementary Materials and Methods.

### Western blot, IP, and Co-IP analyses

Western blot, IP, and Co-IP analyses were conducted as described in a previous study [37]. The detailed methods and information regarding the antibodies used can be found in the Supplementary Materials and Methods.

### RNA extraction and quantitative real-time polymerase chain reaction assay

The experiments were conducted following the standard protocols routinely used in our laboratory [38]. Details of methods and information are provided in the Supplementary Materials and Methods.

### Lentiviral production and infection

The expression plasmid and packaging plasmids (psPAX2 and pMD2.G) were cotransfected into HEK293T cells to produce lentiviral vectors. The process for generating stable cell lines using lentivirus is outlined in the Supplementary Materials.

### Cell viability assay

Cell Counting Kit-8 assays were conducted following the standard protocols routinely used in our laboratory [39].

### Epigenetic compound library screen

A total of 765 epigenetic compounds were obtained from MCE (HY-L005); 786-O cells (3 × 10^3^ per well) were seeded into 96-well plates and, the next day, treated with 0.2 μM RSL3 along with either dimethyl sulfoxide or a compound (10 μM). Following 12 h of incubation, cell relative viability was assessed using the Cell Counting Kit-8 assay.

### GST pull-down assay

GST pull-down assays were conducted following the standard protocols routinely used in our laboratory [40].

### Lipid peroxidation assay

Lipid peroxidation assays were conducted following the standard protocols routinely used in our laboratory [39].

### Mitochondrial membrane potential assay

JC-1 Assay Kit (C2006, Beyotime) was employed to assess mitochondrial membrane potential, which was then analyzed by flow cytometry.

### MDA assay

Following cell lysis, the supernatants were collected, and MDA levels were determined using a lipid peroxidation assay kit (S0131, Beyotime) in accordance with the manufacturer’s protocol.

### Labile iron pool measurement

Cells were seeded into 6-well plates at a density of 1 × 10^6^ cells per well and treated with the specified compounds on the following day. Subsequently, cells were harvested, and intracellular Fe^2+^ levels were measured using a divalent iron assay kit (Abcam, ab83366) following the manufacturer’s instructions.

### MS analysis

Comprehensive details regarding the preparation of ACSL4 samples for methylation analysis using MS can be found in the Supplementary Materials. Liquid chromatography–tandem MS assays were conducted by Applied Protein Technology Co., Ltd. (Shanghai, China).

### Antibody generation and detection

An antibody targeting SDMA-R549-ACSL4 (anti-meR549-ACSL4) was produced to recognize the region surrounding the R549 ADMA site of ACSL4. A synthetic peptide (DENGQ(R-Me2)WFCTGDIGE-C) containing asymmetric dimethylation was utilized for immunizing rabbits. The antibody was developed by the GL Biochem company (Shanghai, China).

### Immunohistochemistry

IHC was performed following the standard streptavidin–peroxidase method, as detailed in a previous study [41]. A comprehensive description of the antibodies used and the IHC evaluation procedures can be found in the Supplementary Materials and Methods.

### Patients and sample collection

Between 2005 and 2008, 307 RCC tissue samples were collected at the Affiliated Hospital of Xuzhou Medical University and used to construct TMA slides. Patient clinical and histopathological data were subsequently obtained from institutional records.

### Animal works

Xenograft tumor models were established using BALB/c nude mice and BALB/c mice aged 6 to 8 weeks. RCC cells were collected, rinsed twice with phosphate-buffered saline, and resuspended in a 1:1 mixture with Matrigel. Each mouse received a subcutaneous injection of 5 × 10^6^ cells in a total volume of 100 μl. In certain experiments, RSL3 was administered intratumorally at a dose of 100 mg/kg twice weekly, while Fer-1 was delivered intraperitoneally at 2 μg/mg daily for 14 consecutive days. For models using BALB/c mice, anti-PD1 antibody (200 μg per dose) was injected intraperitoneally, and the PRMT5i (5 mg/kg) was given orally once every 3 d. Tumors were harvested for further evaluation on day 25 or day 19, depending on the experimental design, following euthanasia of the animals.

### Statistical analysis

Statistical analyses of the TMA slides were conducted using the SPSS 22 software. The relationship between PRMT5 staining and the clinicopathologic features of renal cancer patients was assessed via the χ^2^ test. Kaplan–Meier survival curves and log-rank tests were employed to examine the prognostic significance of PRMT5 expression. Multivariate analysis was carried out using a Cox proportional hazards regression model. A Student *t* test was applied to assess statistical differences between groups, with the significance set at *P* < 0.05. All values are expressed as mean ± SD. Statistical analyses were performed using the GraphPad Prism software.

## Ethical Approval

This study was conducted in compliance with the principles of the Declaration of Helsinki. Informed consent was obtained from all subjects. Ethics approval for human subjects was provided by the Ethics Committee of the Affiliated Hospital of Xuzhou Medical University. Ethics approval for animal work was provided by the Institutional Animal Care and Use Committee of Xuzhou Medical University.

## Data Availability

The data supporting the findings of this study are available from the corresponding authors upon reasonable request.
