# Supplementary material for: PRMT5-Mediated Arginine Methylation of ACSL4 Attenuates Its Stability and Suppresses Ferroptosis in Renal Cancer
Source: Research (Wash D C). 2025 Aug 1;8:0789. doi: 10.34133/research.0789 (PMC12314280; doi:10.34133/research.0789)
Supplement: Supplementary 1 — Supplementary Materials and Methods Figs. S1 to S6 Tables S1 to S3 [file research.0789.f1.docx]

**PRMT5-mediated ACSL4 arginine methylation attenuates its stability and suppresses ferroptosis in renal cancer**

Meng Zhang^1,2#^, Hao Liu^2#^, Rong Yin^3#^, Jiayu Xu^3^, Siqi Fan^1,2^, Xingyou Qian^1^, Menghan Cao^1,2^, Shu Li^4^, Ao Zhang^4^, Guodong Chen^4^, Hongmei Yong^3*^, Zhongwei Li^1,4,5*^ and Jin Bai^1,2,6*^

^1^Cancer Institute, Xuzhou Medical University, Xuzhou, Jiangsu, China;

^2^Centre of Clinical Oncology, the Affiliated Hospital of Xuzhou Medical University, Xuzhou, Jiangsu, China;

^3^Department of Oncology, The Affiliated Huai'an Hospital of Xuzhou Medical University and The Second People's Hospital of Huai'an, Huai’an, Jiangsu, China;

^4^Laboratory of Epigenetic Regulation in Molecular Medicine, School of Basic Medical Sciences, Wannan Medical College, Wuhu, Anhui, China;

^5^Anhui Province Key Laboratory of Basic Research and Transformation of Age-related Diseases, Wannan Medical College, Wuhu, Anhui, China;

^6^Jiangsu Center for the Collaboration and Innovation of Cancer Biotherapy, Cancer Institute, Xuzhou Medical University, Xuzhou, Jiangsu, China.

**Authorship notes:** #These authors contributed equally to this work.

**Running title**: PRMT5 suppresses renal cancer cells ferroptosis.

***Correspondence Authors:**

Jin Bai, Cancer Institute, Xuzhou Medical University. 209 Tongshan Road, Xuzhou, 221004, Jiangsu Province, China. E-mail: bj@xzhmu.edu.cn.

Zhongwei Li, Laboratory of Epigenetic Regulation in Molecular Medicine, School of Basic Medical Sciences, Wannan Medical College. Wenchang West Road No.22, Wuhu Higher Education Park, Wuhu City, 241002, Anhui Province, China. E-mail: lizw074@wnmc.edu.cn.

Hongmei Yong, Department of Oncology, The Second People’s Hospital of Huai’an, 62 South Huaihai Road, Huaian, Jiangsu, 223001, China. E-mail: Ha2280@126.com.

**Conflict of interest:** No potential conflicts of interest were disclosed by the authors.

**Supplementary Materials and Methods**

**Cell culture and treatment**

10% FBS was added to the RPMI-1640 media when cultivating 786-O and Renca cells. ACHN and HEK293T cells were grown in DMEM supplemented with 10% FBS.

The small interfering RNAs (siRNA, 50 nM) against human PRMT5 were transfected into the RCC cells with SilenFect reagent (Thermo Fisher Scientific Inc., USA), while non-specific siRNA was used as negative controls. All siRNAs were purchased from Gene pharma Technology (Shanghai, China).

**Western blot**

Total protein from cells was extracted by using RIPA lysis buffer and was qualified by using a BCA detecting kit (Keygen, Nanjing, China). Proteins samples were subjected to 10% SDS-PAGE and transferred onto a PVDF membrane, and then incubated with specific antibody at 4℃ overnight, respectively. The next day, the membranes were incubated with secondary antibodies included HRP-goat anti-mouse, HRP-goat anti-rabbit (ABclonal) at room temperature for 1h. Protein bands were detected on Tanon 5200. Automatic chemiluminescence imaging analysis system using ECL reagent (Tanon, Shanghai, China).

**Immunoprecipitation and co-immunoprecipitation**

Cell lysates were obtained by incubating the cells in lysis bufer (50mM Tris–HCl, pH8.0; 0.2% NP-40, 150mM; NaCl, 2mM, EDTA and protease inhibitor cocktail) for 20min at 4℃, followed by centrifugation at 14,000 g for 15min at 4℃. Overall, 5% whole-cell extracts were used for input. The rest of the protein extracts were incubated with 2μg control or specific antibodies overnight at 4℃. Then, 10μL of Anti-Myc Nanobody Agarose beads / Anti-Flag Nanobody Agarose beads (KT HEALTH KTSM1306/KTSM1308) were added with further incubation at 4℃ for 2h. Beads were then washed five times using the cold lysis buffer. The immunoprecipitates were boiled with 2×SDS-PAGE loading bufer, separated on SDS-PAGE gels, followed by immunoblotting with various antibodies indicated.

**Antibodies and reagents**

The specific antibodies and reagents used in this study were as follows: Antibody against Tubulin was used as control (Proteintech, Cat #10068-1-AP). PRMT5 (Proteintech, Cat #18436-1-AP), ACSL4 (Proteintech, Cat #66617-1-Ig), CDK1 (Proteintech, Cat #67575-1-Ig), PRDX1 (Proteintech, Cat #66820-1-Ig), SDMA (Cell Signaling Technology, Cat#13222), PARP1 (Proteintech, Cat #66520-1-Ig), Ub (Cell Signaling Technology, Cat#3933) FSP1 (Proteintech, Cat #68049-1-Ig) were used for Western blot assays. The anti-SDMA-R549-ACSL4 (anti-meR549-ACSL4) antibody was raised against the region near R549 symmetric dimethylarginine site of ACSL4. The symmetric di-methylated synthetic peptide [DENGQ(R-Me2,symmetrical)WFCTGDIGE-Cys] was used for immunization in the rabbit. The antibody was generated in Gl biochem company (Shanghai, China). GSK3326595 (Selleck, S8664) was purchased from Selleck (Houston, Texas, USA). The epigenetic compound library was purchased from MCE (HY-L005).

**RNA extraction and quantitative real-time PCR (qRT-PCR) assay**

Total RNA from cell and tissue samples was isolated using TRIzol Reagent (Invitrogen, USA) according to the manufacturer’s protocols, and cDNA was synthesized using the HiScript 1st Strand cDNA Synthesis Kit (Vazyme Biotech, Nanjing, China). Quantitative realtime PCR was carried out on ABI-7500 using UltraSYBR One Step RT-qPCR Kit (CWBIO, Beijing, China). The relative mRNA expression levels were normalized to and GAPDH.

**Identification of ACSL4 methylation sites by mass spectrometry**

To identify in vivo methylation sites of ACSL4, HEK293T cells were co-transfected with FLAG-ACSL4 and MYC-PRMT5. The cell lysates were coimmunoprecipitated with an anti-FLAG antibody. The immunoprecipitated FLAG-ACSL4 was subjected to SDS-PAGE. And the bands corresponding to ACSL4 were subjected to in-gel trypsin digestion. The labeled peptides were analyzed with Liquid chromatography-tandem mass spectrometry analysis (LC-MS/MS) performed in the APPLIED PROTEIN TECHNOLOGY, Shanghai, China.

**Immunohistochemistry (IHC)**

In IHC assays, heat-induced epitope retrieval was performed with retrieval buffer (EDTA, pH9.0 or citrate, pH6.0). The primary antibodies used in IHC are described as follows: PRMT5 antibody (18436-1-AP, Proteintech), meR549-ACSL4 antibody, Ki-67 antibody (12202, CST), CD3 antibody (17617-1-AP, Proteintech), CD8a antibody (A23081, ABclonal), GZMB antibody (13588-1-AP, Proteintech). Briefly, the detailed IHC assessment method of TMAs was performed as the staining scores of PRMT5 and meR549-ACSL4 were evaluated via combining the percentage of cells with the staining intensity and the IRS (immunoreactivity score, IRS) by three pathologists separately. The intensity of PRMT5 and meR549-ACSL4 immunostaining was scored as 0–3 (0, negative; 1, weak; 2, moderate; 3, strong); the percentage of immunoreactivity cells was graded as 1 (0–25%), 2 (26–50%), 3 (51–75%), and 4 (76–100%).

**Supplementary Figure Legends**


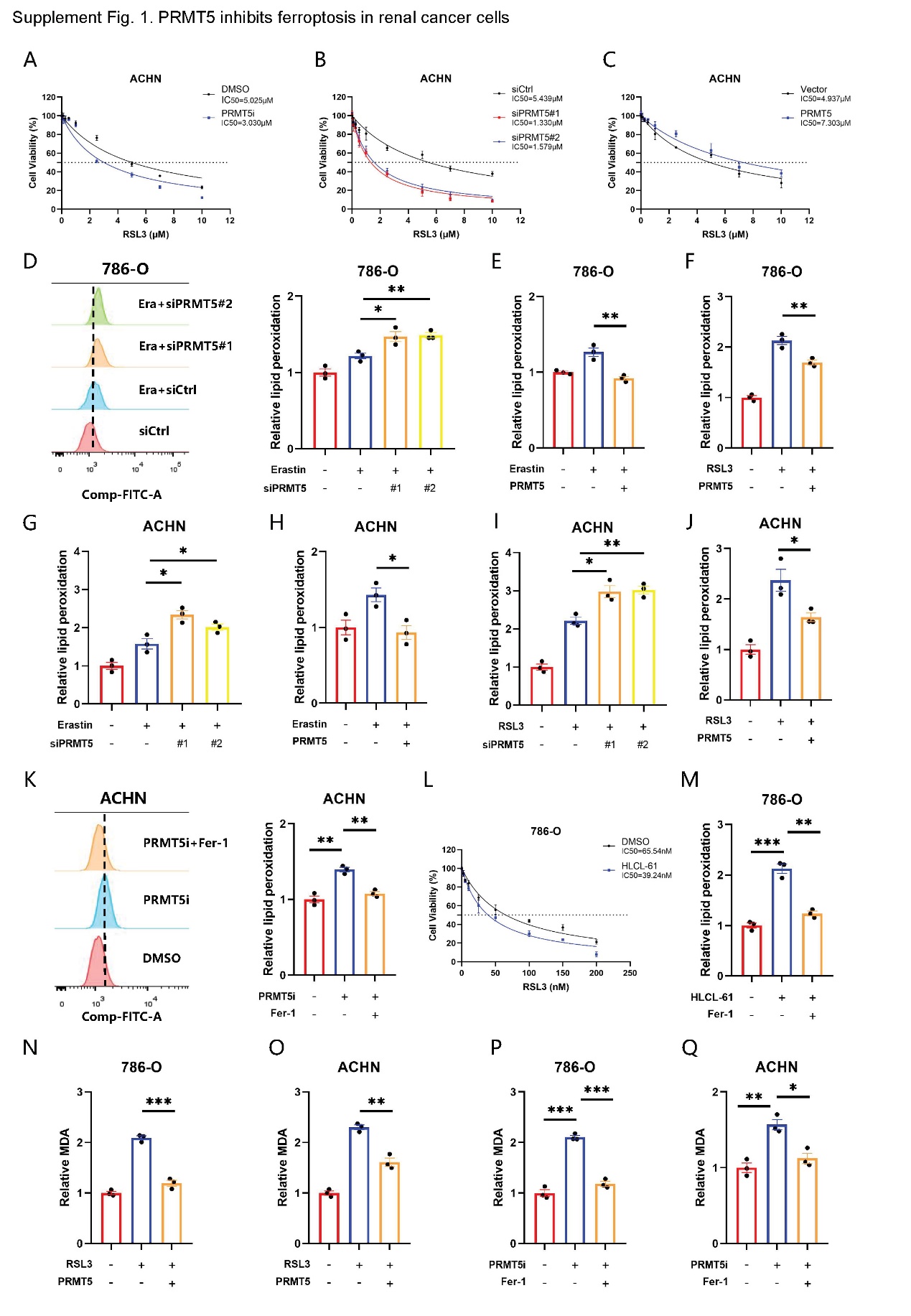


**Supplement Fig. 1. PRMT5 inhibits ferroptosis in** **renal cancer cells**

(A-C) After treated with PRMT5i (A), PRMT5 knockdown (B) or overexpressed (C), ACHN cells were treated with RSL3 for 12 hours. The cell viability was examined using the CCK-8 assay. (D-F) PRMT5 knockdown or overexpressed 786-O cells were treated with 10μM erastin (D, E) or 0.2 μM RSL3 (F) for 12 hours to assess lipid peroxidation. (G-J) PRMT5 knockdown or overexpressed ACHN cells were treated with 40μM erastin (G, H) or 2 μM RSL3 (I, J) for 12 hours to determine lipid peroxidation. (K) ACHN cells were treated with 20 μM PRMT5i combination with/without Fer-1 for 12 hours to detect lipid peroxidation. (L-M) After treated with another PRMT5 inhibitor, HLCL-61, 786-O cells were treated with RSL3 for 12 hours. The cell viability (L) and lipid peroxidation (M) were examined. (N, O) After treated with PRMT5i, 786-O (N) and ACHN (O) cells were examined using the MDA assay. (P, Q) After treated with PRMT5 overexpressed, 786-O (P) and ACHN (Q) cells were examined using the MDA assay.


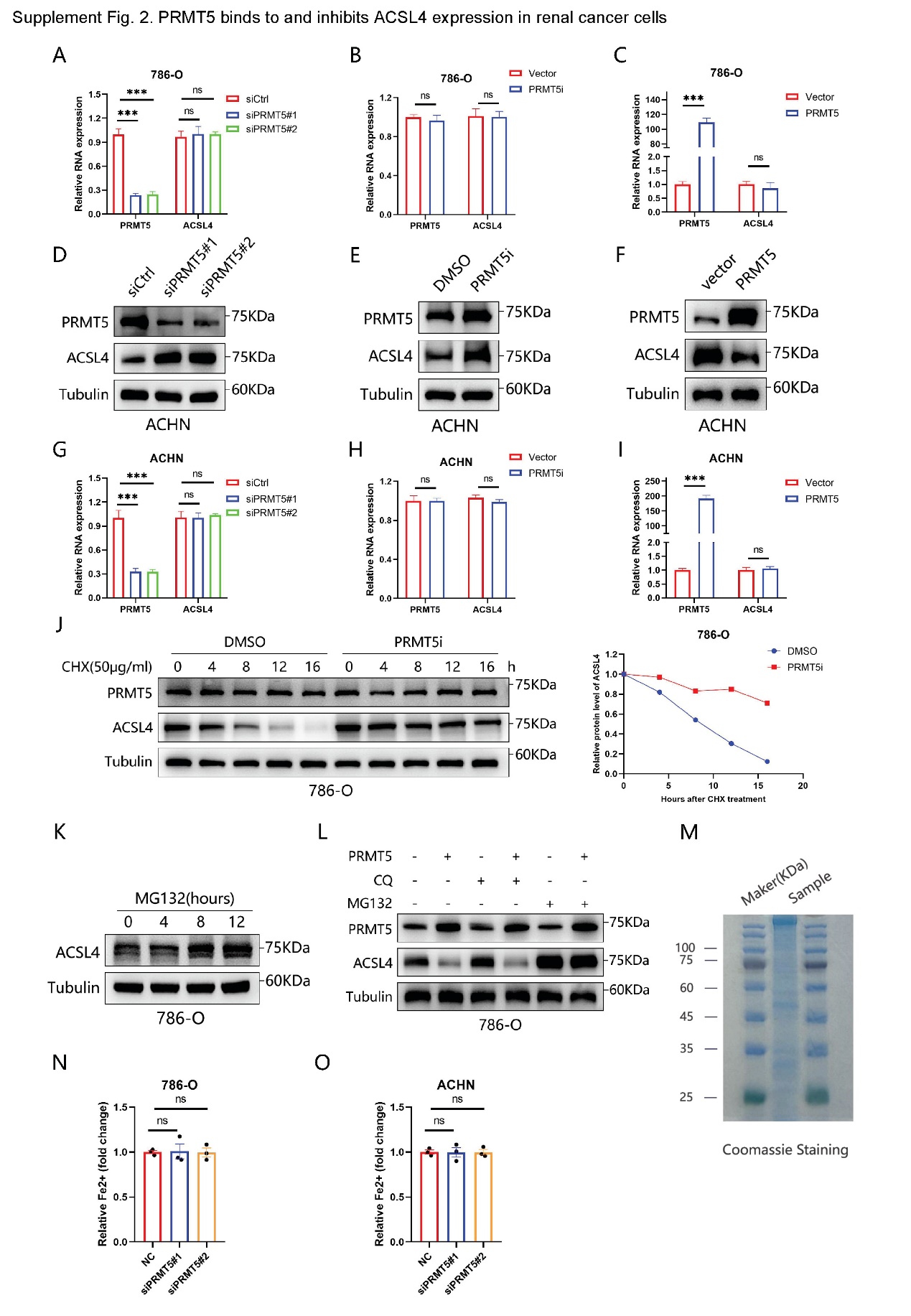


**Supplement Fig. 2. PRMT5 binds to and inhibits ACSL4 expression in renal cancer cells**

(A-C) ACSL4 mRNA levels were analyzed by qRT-PCR experiments in 786-O cells treated by PRMT5 knockdown (A), PRMT5 inhibitor (B) or PRMT5 overexpressed (C). (D-I) ACSL4 protein and mRNA levels were analyzed by Western blot and qRT-PCR experiments in ACHN cells treated by PRMT5 knockdown (D, G), PRMT5 inhibitor (E, H) or PRMT5 overexpressed (F, I). (J) The ACSL4 protein expression was examined by western blot in 786-O cells with/without PRMT5 inhibition following treated by CHX (50 μg/ml) for the specified time. (K) The ACSL4 protein expression was examined by western blot in 786-O cells treated by 10 μM MG132 for the specified time. (L) The PRMT5 and ACSL4 protein expression was examined by western blot in 786-O cells with/without PRMT5 overexpressed following treated by DMSO, 10 μM MG132 or 25 μM CQ for 8 h. (M) IP assay was performed to enrich the PRMT5 protein, followed by Coomassie Brilliant Blue staining, and subsequent verification using mass spectrometry. mRNA, messenger RNA; qRT-PCR, quantitative reverse transcription polymerase chain reaction. (N, O) Cellular divalent iron levels were assayed in the 786-O (N) and ACHN cells (O).


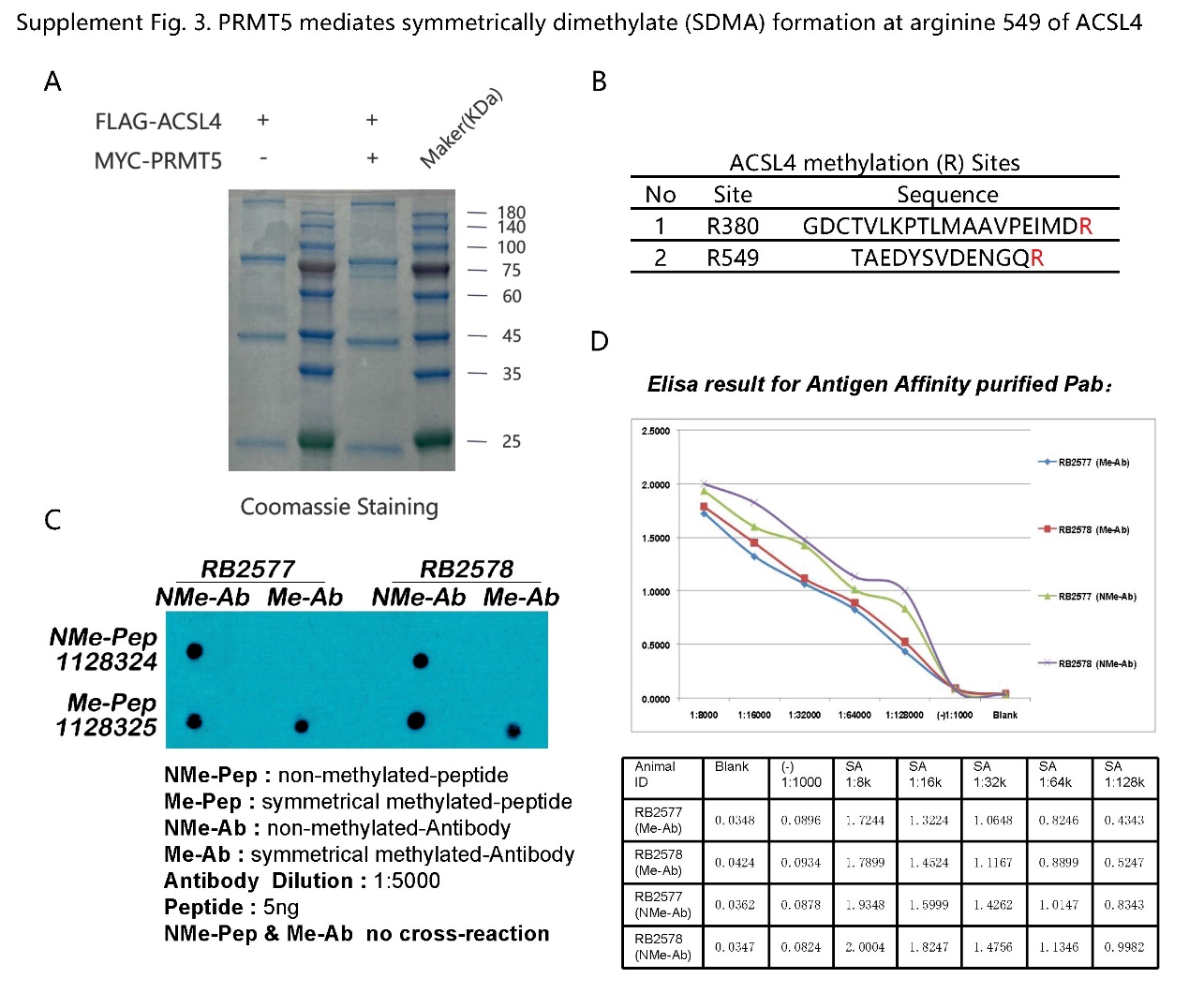


**Supplement Fig. 3. PRMT5 mediates symmetrically dimethylate (SDMA) formation at arginine 549 of ACSL4**

(A) IP assay was performed to enrich the ACSL4 protein, followed by Coomassie Brilliant Blue staining, and subsequent verification using mass spectrometry. (B) MS analysis of ACL4 methylation. The fragmentation of the ACSL4 peptide identified with a dimethylated arginine residue. (C-D) Elisa and Dot blots result for Antigen Affinity purified Pab (antibody) for anti-meR549-ACSL4.


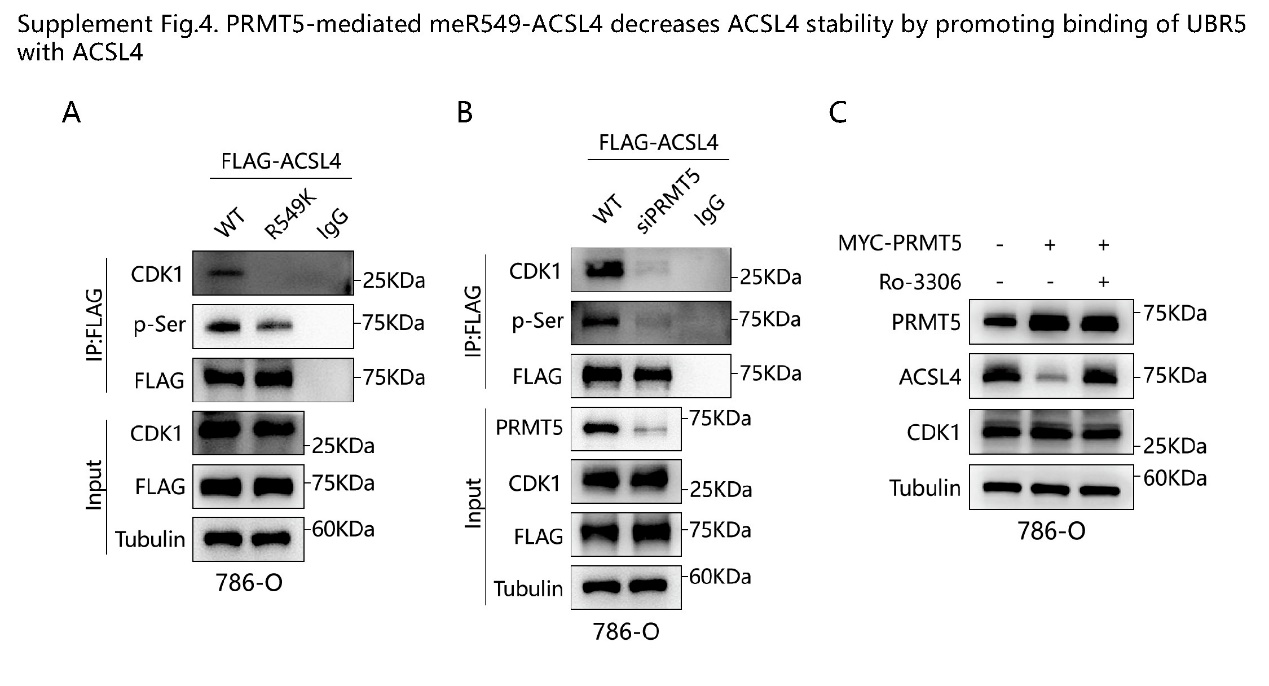


**Supplement Fig.4. PRMT5-mediated meR549-ACSL4 decreases ACSL4 stability by promoting binding of UBR5 with ACSL4**

1. Co-IP was performed to detect ACSL4 interaction with CDK1 and serine phosphorylation levels in ACSL4-WT and ACSL4-R549K mutant 786-O cells. (B) Co-IP was performed to detect ACSL4 interaction with CDK1 and serine phosphorylation levels in 786-O cells with/without PRMT5 knockdown. (C) Western blot analysis of vector and PRMT5 upregulation 786-O cells treated with DMSO CDK1 inhibitor Ro-3306 for 24 h. Protein levels of PRMT5, ACSL4 and CDK1 were detected.


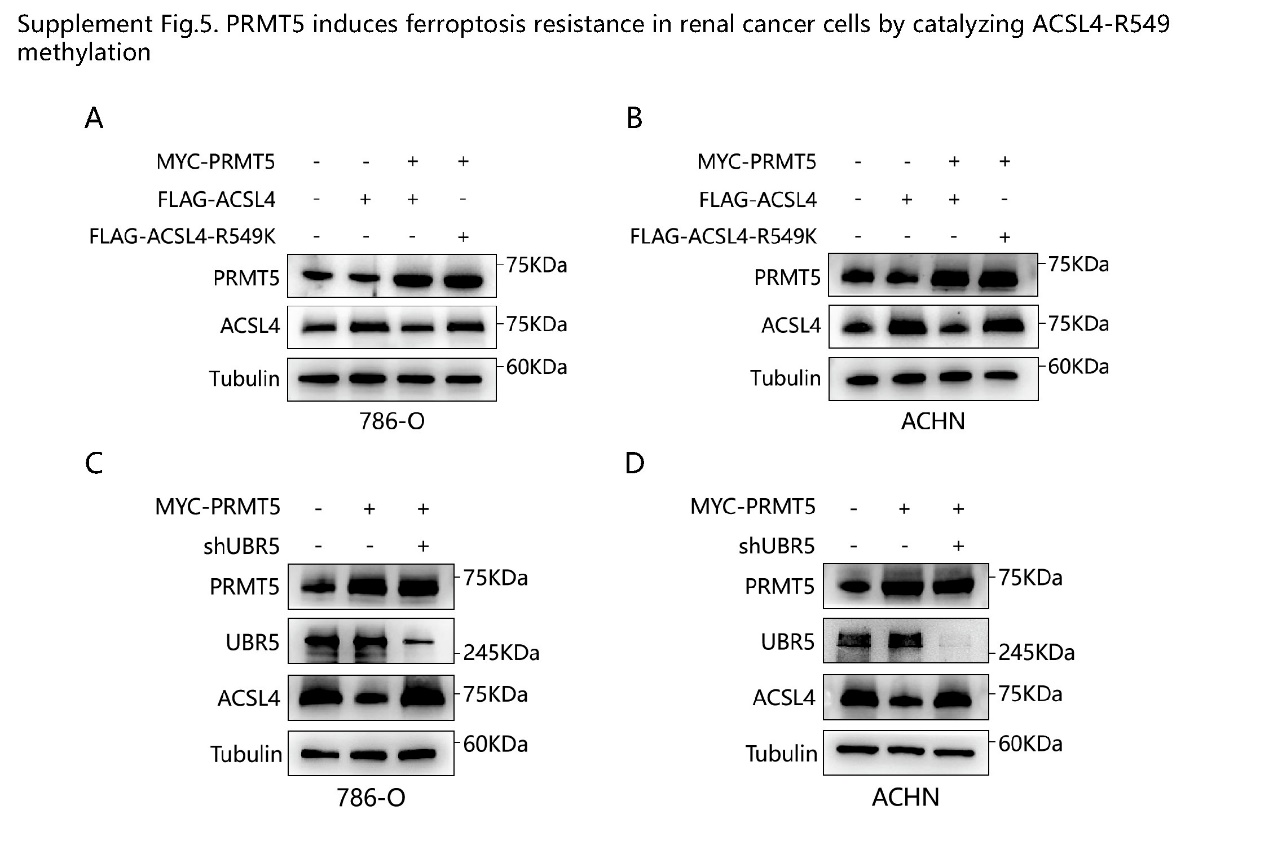


**Supplement Fig.5. PRMT5 induces ferroptosis resistance in renal cancer cells by R549-ACSL4 methylation**

(A, B) After treated with overexpressing ACSL4, overexpressing ACSL4 combination of PRMT5 and overexpressing ACSL4-R549K combination of PRMT5, 786-O (A) and ACHN (B) cells were examined using Western Blot. (C, D) After treated with overexpressing PRMT5 and overexpressing PRMT5 combination of UBR5 knockdown, 786-O (C) and ACHN (D) cells were examined using Western Blot.

**
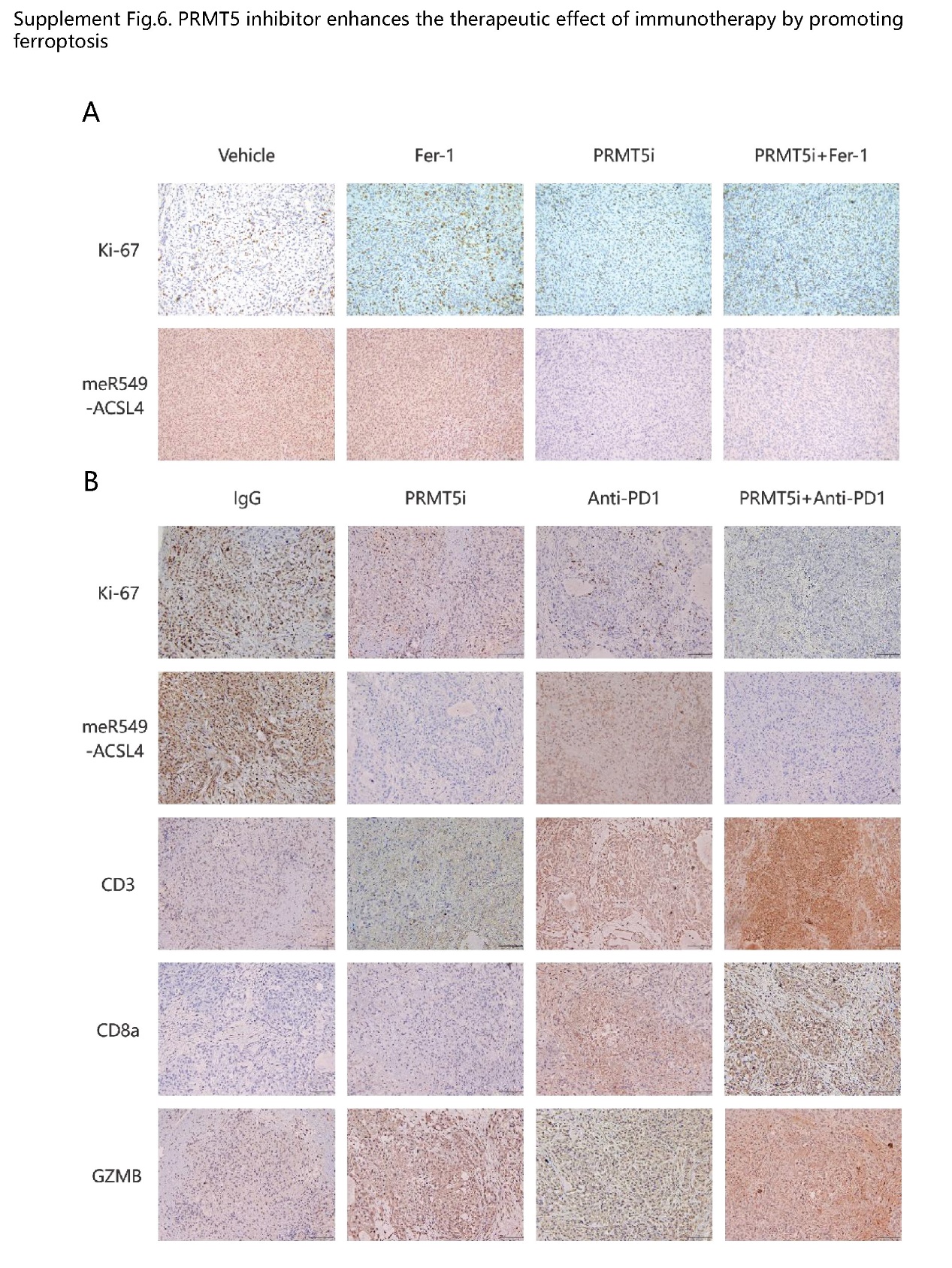
**

**Supplement Fig.6. PRMT5 inhibitor enhances the therapeutic effect of immunotherapy by promoting ferroptosis**

(A-B) Representative images of the IHC staining of Ki-67, meR549-ACSL4, CD3, CD8a and GZMB in the tumor xenografts.

**Table S1: PRMT5 staining and clinicopathological characteristics of renal cancer patients**

| **variables** | **PRMT5 staining** | | | |
| --- | --- | --- | --- | --- |
|  | **Low(%)** | **High(%)** | **Total** | ***P**** |
| **Age** |  |  |  |  |
| ≤ 56 years | 103(75.2) | 34(24.8) | 137 | 0.167 |
| > 56 years | 101(67.8) | 48(32.2) | 149 |  |
| **Tumor size** |  |  |  |  |
| ≤ 7cm | 163(76.2) | 51(23.8) | 214 | 0.002 |
| > 7cm | 41(56.9) | 31(43.1) | 72 |  |
| **Depth of invasion** |  |  |  |  |
| Intra-renal | 152(82.6) | 32(17.4) | 184 | <0.001 |
| Extra-renal | 52(51.0) | 50(49.0) | 102 |  |
| **Lymph node metastasis** |  |  |  |  |
| Negative | 181(74.5) | 62(25.5) | 243 | 0.005 |
| positive | 23(53.5) | 20(46.5) | 43 |  |
| **Distant metastasis** |  |  |  |  |
| Negative | 172(78.9) | 46(21.1) | 218 | <0.001 |
| positive | 32(47.1) | 36(52.9) | 68 |  |
| **Urinary system diseases** |  |  |  |  |
| Negative | 192(71.9) | 75(28.1) | 267 | 0.415 |
| positive | 12(63.2) | 7(36.8) | 19 |  |

*Two sided Pearson Chi-Square tests.

Some cases were not available for the information.

**Table S2: Univariate Cox proportional regression analysis on 5-year overall survival and disease-free survival of renal cancer patients**

| **Variable*** | **Overall survival** | | | **Disease-free survival** | | |
| --- | --- | --- | --- | --- | --- | --- |
|  | **Hazard ratio** | **95％CI^†^** | ***P**** | **Hazard ratio** | **95％CI^†^** | ***P**** |
| **PRMT5** |  |  |  |  |  |  |
| Low | 1.000 |  | <0.001 | 1.000 |  | <0.001 |
| High | 2.209 | 1.601-3.049 |  | 2.306 | 1.608-3.306 |  |
| **Age** |  |  |  |  |  |  |
| ≤ 56 years | 1.000 |  | 0.614 | 1.000 |  | 0.694 |
| > 56 years | 1.074 | 0.814-1.417 |  | 1.061 | 0.791-1.422 |  |
| **Tumor size** |  |  |  |  |  |  |
| ≤ 7cm | 1.000 |  | 0.002 | 1.000 |  | 0.015 |
| > 7cm | 1.633 | 1.206-2.212 |  | 1.503 | 1.083-2.084 |  |
| **Depth of invasion** |  |  |  |  |  |  |
| Intra-renal | 1.000 |  | <0.001 | 1.000 |  | <0.001 |
| Extra-renal | 2.061 | 1.551-2.739 |  | 1.908 | 1.409-2.583 |  |
| **Lymph node metastasis** |  |  |  |  |  |  |
| Negative | 1.000 |  | <0.001 | 1.000 |  | 0.005 |
| positive | 2.913 | 1.903-4.467 |  | 2.143 | 1.258-3.650 |  |
| **Distant metastasis** |  |  |  |  |  |  |
| Negative | 1.000 |  | <0.001 | 1.000 |  | <0.001 |
| positive | 3.453 | 2.472-4.824 |  | 3.228 | 2.219-4.697 |  |
| **Urinary system diseases** |  |  |  |  |  |  |
| Negative | 1.000 |  | 0.884 | 1.000 |  | 0.340 |
| positive | 0.959 | 0.546-1.683 |  | 0.721 | 0.369-1.410 |  |

**P* values are from Log-rank test.

^†^CI: confidence interval.

**Table S3: Multvariate Cox regression analysis on 5-year overall survival and disease-free survival of renal cancer patients**

| **Variable*** | **Overall survival** | | | **Disease-free survival** | | |
| --- | --- | --- | --- | --- | --- | --- |
|  | **Hazard ratio** | **95％CI^†^** | ***P**** | **Hazard ratio** | **95％CI^†^** | ***P**** |
| PRMT5 | 1.665 | 1.146-2.417 | 0.007 | 1.845 | 1.229-2.759 | 0.003 |
| Age | 0.914 | 0.688-1.214 | 0.534 | 0.927 | 0.687-1.252 | 0.623 |
| Tumor size | 1.153 | 0.800-1.662 | 0.444 | 1.199 | 0.815-1.764 | 0.358 |
| Depth of invasion | 1.465 | 0.996-2.156 | 0.052 | 1.355 | 0.911-2.013 | 0.133 |
| Lymph node metastasis | 1.216 | 0.697-2.122 | 0.490 | 0.931 | 0.466-1.861 | 0.839 |
| Distant metastasis | 2.858 | 1.820-4.488 | <0.001 | 2.969 | 1.798-4.901 | <0.001 |
| Urinary system diseases | 1.040 | 0.584-1.850 | 0.895 | 0.871 | 0.443-1.715 | 0.690 |

*Coding of variables: PRMT5 was coded as 1 (low), and 2 (high). Age was coded as 1 (≤ 56 years), and 2 (> 56 years). Tumor size was coded as 1 (≤ 7cm), and 2 (> 7cm). Depth of invasion was coded as 1 (intra-renal), and 2 (extra-renal). Lymph node metastasis was coded as 1 (negative), and 2 (positive). Distance metastasis was coded as 1 (negative), and 2 (positive). Urinary system diseases was coded as 1 (negative), and 2 (positive).

^†^CI: confidence interval.
